# Components of Adolescent Behavioural Interventions With Eating Disorder Outcomes: Systematic Review With Intervention Mapping

**DOI:** 10.1111/ijpo.70074

**Published:** 2025-12-10

**Authors:** Natalie B. Lister, Rabia Khalid, Isabelle R. Jardine, Samantha Pryde, Hannah Melville, Anna L. Seidler, Kylie E. Hunter, Amy L. Ahern, Louise A. Baur, Caroline Braet, Sarah P. Garnett, Andrew J. Hill, Sarah Maguire, Dasha Nicholls, Susan J. Paxton, Milan K. Piya, Amanda Sainsbury, Katharine Steinbeck, Denise E. Wilfley, Kelly Cooper, Genevieve Dammery, Alicia M. Grunseit, Faith Anne N. Heeren, Rebecca A. Jones, Theodore K. Kyle, Fiona Quigley, Molly Robbins, Jacqlyn Yourell, Melanie K. Bean, Maxine P. Bonham, Kerri N. Boutelle, Michelle I. Cardel, Katherine E. Darling, Aimee L. Dordevic, Dawn M. Eichen, Leonard H. Epstein, Andrea B. Goldschmidt, Elissa Jelalian, Mara Cristina Lofrano‐Prado, Tiffany Naets, Wagner L. Prado, Hanna F. Skjåkødegård, Yngvild Sørebø Danielsen, Richard I. Stein, Marian Tanofsky‐Kraff, Annelies Van Eyck, Alaina P. Vidmar, Jack A. Yanovski, Brittany J. Johnson, Hiba Jebeile, the EDIT Collaboration, Natalie B. Lister, Hiba Jebeile, Anna L. Seidler, Brittany J. Johnson, Rabia Khalid, Hannah Melville, Sol Libesman, Ruth Tredinnick, Samantha Pryde, Caitlin M. McMaster, Kylie E. Hunter, Isabelle R. Jardine, Sasha J. Lorien, Amy L. Ahern, Lisa Askie, Louise A. Baur, Caroline Braet, Kelly Cooper, Genevieve Dammery, Sarah P. Garnett, Rebecca Golley, Alicia M. Grunseit, Faith Anne N. Heeren, Andrew J. Hill, Rebecca A. Jones, Theodore K. Kyle, Sarah Maguire, Dasha Nicholls, Susan J. Paxton, Milan K. Piya, Fiona Quigley, Molly Robbins, Amanda Sainsbury, Katharine Steinbeck, Denise E. Wilfley, Jacqlyn Yourell, Rachel D. Barnes, Melanie K. Bean, Kristine Beaulieu, Maxine P. Bonham, Kerri N. Boutelle, Caroline Braet, Braulio Branco, Simona Calugi, Michelle I. Cardel, Kelly M. Carpenter, Hoi Lun Cheng, Riccardo Dalle Grave, Katherine E. Darling, Brenda Davy, Marcelo Demarzo, Aimee L. Dordevic, Dawn M. Eichen, Leonard H. Epstein, Andrea B. Goldschmidt, Amy Gross, Faith Anne N. Heeren, Anja Hilbert, Katrijn Houben, Erica M. Howes, Elissa Jelalian, Natalie B. Lister, Mara Cristina Lofrano‐Prado, Corby Martin, Anne McTiernan, Janell Mensinger, Tiffany Naets, Dasha Nicholls, Carly Pacanowski, Stephanie Partridge, Wagner L. Prado, Sofia Marques Ramalho, Jaythani Raman, Hollie A. Raynor, Kyung E. Rhee, Elizabeth Rieger, Eric Robinson, Amanda Sainsbury, Vera Salvo, Brian M. Shelley, Nancy E. Sherwood, Sharon A. Simpson, Hanna F. Skjakodegard, Evelyn Smith, Yngvild Sørebø Danielsen, Richard I. Stein, Marian Tanofsky‐Kraff, Rachael W. Taylor, Annelies Van Eyck, Krista A. Varady, Alaina P. Vidmar, Victoria Whitelock, Denise E. Wilfley, Jack A. Yanovski

**Affiliations:** ^1^ Children's Hospital Westmead Clinical School The University of Sydney Westmead New South Wales Australia; ^2^ Charles Perkins Centre The University of Sydney, Camperdown New South Wales Australia; ^3^ College of Nursing and Health Sciences Caring Futures Institute, Flinders University Adelaide South Australia Australia; ^4^ National Health and Medical Research Council Clinical Trials Centre The University of Sydney Sydney New South Wales Australia; ^5^ Department of Child and Adolescent Psychiatry University Medical Centre Rostock Rostock Germany; ^6^ German Center for Child and Adolescent Health (DZKJ), Partner Site Greifswald/Rostock Rostock Germany; ^7^ MRC Epidemiology Unit University of Cambridge Cambridge Cambridgeshire UK; ^8^ The Children's Hospital at Westmead, Weight Management Services Westmead New South Wales Australia; ^9^ Department of Developmental, Personality and Social Psychology Ghent University Ghent Belgium; ^10^ The Children's Hospital at Westmead, Kids Research Westmead New South Wales Australia; ^11^ School of Medicine University of Leeds Leeds UK; ^12^ InsideOut Institute for Eating Disorders The University of Sydney Camperdown New South Wales Australia; ^13^ Imperial College London, Division of Psychiatry, National Institute for Health Research Applied Research Collaboration London UK; ^14^ School of Psychology and Public Health La Trobe University Melbourne Victoria Australia; ^15^ School of Medicine Western Sydney University Campbelltown New South Wales Australia; ^16^ Campbelltown and Camden Hospitals Campbelltown New South Wales Australia; ^17^ School of Human Sciences The University of Western Australia Crawley Western Australia Australia; ^18^ School of Medicine Washington University in St. Louis St Louis Missouri USA; ^19^ The Weight Issues Network Sydney New South Wales Australia; ^20^ Department of Health Outcomes and Biomedical Informatics University of Florida College of Medicine Gainesville Florida USA; ^21^ WeightWatchers International, Inc. New York New York USA; ^22^ ConscienHealth Pittsburgh Pennsylvania USA; ^23^ Institute of Nursing and Health Research Ulster University Newtownabbey Co. Antrim UK; ^24^ College of Psychology Nova Southeastern University Davie Florida USA; ^25^ Fit Minded Inc. Phoenix Arizona USA; ^26^ Department of Pediatrics, School of Medicine Children's Hospital of Richmond at Virginia Commonwealth University Richmond Virginia USA; ^27^ Department of Nutrition, Dietetics & Food, Faculty of Medicine, Nursing & Health Sciences Monash University Notting Hill Victoria Australia; ^28^ Department of Pediatrics University of California San Diego San Diego California USA; ^29^ Department of Psychiatry and Human Behavior Alpert Medical School of Brown University Providence Rhode Island USA; ^30^ Weight Control and Diabetes Research Center The Miriam Hospital Providence Rhode Island USA; ^31^ Department of Pediatrics, Jacobs School of Medicine, and Biomedical Sciences University at Buffalo Buffalo New York USA; ^32^ Department of Psychiatry University of Pittsburgh School of Medicine Pittsburgh Pennsylvania USA; ^33^ Department of Psychology & Kinesiology California State University San Bernardino California USA; ^34^ Department of Health Care Odisee University of Applied Sciences, Nutrition and Dietetics Ghent Belgium; ^35^ Department of Kinesiology California State University San Bernardino California USA; ^36^ Children and Youth Clinic Haukeland University Hospital Bergen Vestland Norway; ^37^ Counseling on Eating Disorders ROS Bergen Vestland Norway; ^38^ Department of Clinical Psychology University of Bergen Bergen Vestland Norway; ^39^ Regional Department of Eating Disorders Haukeland University Hospital Bergen Vestland Norway; ^40^ Department of Medical and Clinical Psychology, School of Medicine Uniformed Services University of the Health Sciences Bethesda Maryland USA; ^41^ Section on Growth and Obesity, Division of Intramural Research, National Institutes of Health Eunice Kennedy Shriver National Institute of Child Health and Human Development Bethesda Maryland USA; ^42^ Laboratory of Experimental Medicine and Pediatrics University of Antwerp Antwerp Antwerp Belgium; ^43^ Department of Pediatrics Antwerp University Hospital Antwerp Belgium; ^44^ Children's Hospital Los Angeles and Keck School of Medicine, Department of Pediatrics University of Southern California California Los Angeles USA; ^45^ Center for Endocrinology, Diabetes and Metabolism California Los Angeles USA

**Keywords:** behaviour change techniques, disordered eating, obesity, obesity treatment, overweight

## Abstract

**Objective:**

To understand delivery features and intervention strategies of adolescent weight management interventions which may influence eating disorder risk.

**Methods:**

Systematic searches in four databases and two trial registries to identify randomised controlled trials in adolescents with overweight/obesity measuring eating disorder risk pre‐ and post‐intervention. Delivery features and intervention strategies were coded from published descriptions using a project‐specific codebook, validated by trial investigators and narratively synthesised.

**Results:**

Of 11 860 records screened, 23 trials, with 54 intervention arms, were included in the analysis. Most interventions focused on weight loss and maintenance (54%) and were informed by a cognitive behavioural framework (43%). Interventions commonly targeted an individual with a support person (70%). Median intervention duration was 26 weeks, with weekly (35%) or staged (e.g., weekly, then monthly) visit (41%) frequency. Interventions had a mean (SD) of 30 (16.1) intervention strategies. Most included healthy eating education (89%), physical activity education (89%) and problem‐solving barriers to dietary change (80%). Few included mental health strategies (17%). Interventions included ‘dietary prescription’ (65%), and 78% promoted ‘healthful/helpful eating behaviours’.

**Conclusion:**

Weight management interventions are complex and vary in delivery approach and strategies used to change behaviors. Characterising interventions is a critical first step to understanding how weight management interventions' influence eating disorder risk.

## 1 Introduction

Behavioural weight management includes multicomponent interventions focusing on diet, physical activity and psychosocial components (e.g., managing stress). The 2023 American Academy of Paediatrics (AAP) clinical practice guidelines for the evaluation and treatment of children and adolescents with obesity recommend Intensive Health Behaviour and Lifestyle Treatment for children 6 years and older [1]. The guidelines also recommend weight loss pharmacotherapy and/or metabolic and bariatric surgery for older adolescents or those with severe obesity (Body Mass Index (BMI) ≥ 120% of the 95th percentile for age and sex); however, these more intensive treatment options should be considered as *an adjunct to* health behaviour and lifestyle treatment. Thus, behavioural weight management remains *foundational to the treatment* of obesity in adolescence both as a stand‐alone and as part of an adjunctive approach [2].

Youth with high body weight are at elevated risk for eating disorders [3], and naturalistic observational studies have demonstrated that dietary restraint and restriction are prospective risk factors for these conditions [4]. Thus, there are ongoing concerns that the provision of obesity treatment, and the use of specific components of some treatment approaches (e.g., energy restriction, self‐monitoring of diet or weight) will exacerbate or induce eating disorder symptoms [5]. Current evidence suggests that structured, professionally delivered weight management interventions under clinical trial conditions do not increase eating disorder risk in most adolescents with obesity [6, 7]. However, certain unknown sub‐groups of adolescents may be at risk of worsening symptoms. Limitations with current literature that require further exploration include sub‐group effects, how change in eating disorder risk should be measured, and effects of withdrawing from an intervention [8]. The Eating Disorders In weight‐related Therapy (EDIT) Collaboration is examining the impact of behavioural weight management on eating disorder risk. EDIT brings together researchers, clinicians and people with lived experience of obesity and/or eating disorders. The rationale and study design for the Collaboration are published elsewhere [9]. To address an important gap in the literature, the EDIT Collaboration will be systematically reviewing and collating all available data from randomised controlled trials (RCTs) reporting on eating disorder risk following behavioural weight management. Accordingly, we hypothesise that components of behavioural weight management interventions available to individuals will vary, and this may have important implications for eating disorder risk.

A key objective of the EDIT Collaboration is to understand how components of behavioural weight management interventions influence eating disorder risk. These interventions are typically multicomponent, including strategies related to diet, physical activity, related health behaviours (e.g., sleep), and psychosocial components, but are also delivered using approaches that vary by mode of delivery (e.g., online or face to face; individual or group) and intervention intensity (i.e., frequency, total dose and duration). The systematic examination of intervention components that may contribute to the risk of eating disorder symptoms is yet to be explored. Previous systematic reviews have demonstrated that certain components of weight management interventions may be more effective for improving weight status [10, 11]. For example, some behaviour change techniques (e.g., goal setting, stimulus control, self‐monitoring of outcomes of behaviour, social reward and social support) may increase intervention effectiveness [10]. An earlier review found that calorie (kilojoule) counting, contact with a dietitian and the use of behaviour change techniques were associated with greater weight loss in adults enrolled in multicomponent behavioural weight management programs [12]. Such research has provided useful insight into the tailoring of weight management interventions to improve effectiveness. However, no such evidence synthesis has examined the *safety* of behavioural weight management components in relation to risk factors for the development of eating disorders. For example, the restriction of energy intake may be prescribed for weight management, yet dietary restriction is a predictor for eating disorder development in community samples [13]. Similarly, tracking of weight loss, caloric intake and/or exercise, may be helpful for weight loss maintenance, but it may also drive an unhelpful focus on weight, shape and eating. Further, life stage may also contribute to the differential effects of certain components, with a recent evidence map showing gaps in clinical guidelines for adolescents compared to adults [14]. It is clear that several behavioural strategies used as part of weight management overlap with features addressed in the treatment of disordered eating and eating disorders and that further research is needed to understand these potential conflicts [5, 15].

Detailed mapping of intervention components (i.e., delivery features and intervention strategies) of behavioural weight management trials is an important step in understanding how weight management trials may increase or decrease eating disorder risk. The aim of the present review was to describe the delivery features and intervention strategies of behavioural weight management interventions that measure eating disorder outcomes in adolescent populations. A secondary aim was to explore the combinations of different types of intervention strategy clusters within such interventions.

## 2 Methods

This systematic review with intervention coding design was prospectively registered on PROSPERO International prospective register of systematic reviews (CRD42021265340), and the protocol was published on Open Science Framework [16]. Changes made to the protocol after registration are listed in Supporting Information S1. This review is one of a series of complementary projects as part of the EDIT Collaboration (www.editcollaboration.com) [9, 17]. Reporting followed the Preferred Reporting Items for Systematic Reviews and Meta‐Analyses (PRISMA) checklist [18].

### 2.1 Eligibility Criteria

Eligible trials included RCTs recruiting adolescents (defined by the World Health Organization as aged 10 to < 19 years [19] at baseline) with overweight or obesity defined as body mass index (BMI) *z*‐score > 1 [20]. Trials needed to include: (1) at least one behavioural weight management intervention aiming to improve a weight‐related outcome (e.g., weight maintenance or weight loss); (2) at least one comparison group (e.g., wait‐list/no‐treatment control, standard care, or alternate intervention); (3) measurement of eating disorder risk at baseline and post‐intervention or follow‐up using a validated assessment tool (e.g., global eating disorder risk score, binge eating). Trials were excluded if interventions were bariatric surgery, post‐surgical interventions, pharmacotherapy or nutrient supplementation, or those targeting secondary or syndromic causes of obesity (e.g., Prader‐Willi Syndrome), any alternate medical condition (e.g., sleep apnoea), eating disorders, or obesity prevention. Trials that targeted adolescents and included parent/adult participants (e.g., using family‐based treatment) were included as adolescent interventions.

### 2.2 Information Sources, Search Strategy and Selection Process

Trials were identified as being eligible for joining the EDIT Collaboration through systematic searches of the electronic databases MEDLINE, Embase, PsycINFO and Scopus from inception to March 2022, and clinical trial registries (ClinicalTrials.gov and the World Health Organization's International Clinical Trials Registry Platform Search Portal [21]) in May 2022. The search string for MEDLINE is presented in Supporting Information S2. Two reviewers independently screened records by title/abstract and full text in Covidence online software (Veritas Health Innovation Ltd., Australia). Discrepancies were resolved by discussion or with a third reviewer. Additional eligible trials were identified through handsearching and professional networks.

### 2.3 Data Collection

One reviewer extracted study characteristics, such as intervention name, location, participant characteristics, number of intervention arms and year of recruitment, which were verified by a second reviewer. Published protocols and manuscripts were the primary data source. Where a secondary outcome paper was identified in the systematic search, the protocol and primary outcome papers were sourced, where applicable, for use in coding. Trial outcome measures, and risk of bias assessments will be reported in a complementary review using individual participant data [17].

### 2.4 Coding Framework

Development and pilot testing of the intervention deconstruction methodology is presented elsewhere [22]. In brief, a detailed codebook was developed through an iterative process with the research team and a range of stakeholders including international researchers, clinicians and those with lived experience of obesity and/or eating disorders. The codebook included a definition for each unique code and any coding assumptions (e.g., if strategy *x* is coded, also code strategy *y*) (Supporting Information S3). One intervention strategy was added since publishing the protocol (‘Language’: weight‐focused or health‐focused language, or a combination) [16].

Delivery features are defined as ‘a broad number of intervention characteristics that relate to how an intervention is delivered’ [23]. Delivery features were adapted from the Template for Intervention Description and Replication (TIDieR) checklist [24], including the ‘Why’ (overarching goal, psychological theory or framework); ‘Target population/recipient of the intervention’ (age group, weight category, support); ‘What’ (materials provided, procedures used, outcome measures); ‘Who’ (background of interventionist and training for intervention administration); ‘How’ (delivery mode, individual/group); ‘Where’ (intervention setting); ‘When and how much’ (intervention dose and post‐intervention support; tailoring; modifications); and ‘How well’ (fidelity). Categories under each delivery feature cluster were developed for this project drawing on relevant examples from published coding frameworks for child obesity prevention trials [23] and the Human Behaviour Change Project ontologies [25] and refined through the aforementioned pilot testing and stakeholder feedback [22, 26].

Intervention strategies are the broad term used to describe the behaviour change content of interventions relevant to weight management. The 89 unique intervention strategies (i.e., lowest‐level grouping) were grouped into one of 20 clusters of similar strategies (i.e., mid‐level grouping), under five key categories (i.e., highest‐level grouping) (See Supporting Information S3). The five key categories were: intervention intent, framing and outcomes (2 clusters; 13 strategies); dietary strategies (5 clusters; 27 strategies); eating behaviours/disordered eating (2 clusters; 10 strategies); movement and sleep‐related strategies (6 clusters; 23 strategies); and psychological health‐related strategies (5 clusters; 16 strategies). All strategies were coded by the coding team, except for one strategy (Language) in the ‘Framing of the intervention (communication strategies)’ cluster that was reported by the trialist. This could not be extracted from publications as it related to the language used with participants.

### 2.5 Intervention Deconstruction Procedures

Outcomes of this review were intervention components, namely delivery features and intervention strategies. Two coders (R.K., S.P.) were trained in using the study‐specific coding manual; one was an Accredited Practising Dietitian, and one had a psychology background. Initially active intervention arms were coded in duplicate to establish consistency between the coders. Active interventions (referred to as ‘interventions’ hereon) included usual care and were defined as any intervention other than no treatment or waitlist control arms. After 75% agreement was reached, interventions were coded by one coder. Presence, absence, or maybe presence code for each component was recorded into a REDCap database (Research Electronic Data Capture tools, hosted by the University of Sydney) [27, 28], per intervention arm. Regular meetings were held between the coding team including the two coders and two senior researchers (N.L., B.J.J.; both dietitians, one with experience in weight management trials and one in intervention coding methods) to discuss any areas of uncertainty and to ensure consistency in coder interpretation.

Finally, all trial investigators were invited to validate coding via video meeting or email. The validation process focused on clarifying any ‘maybe’ codes and confirming other codes. Revised codes were reflected in the final REDCap database. Any disputes raised by trial investigators were discussed and resolved by the study coding team. Agreement of coding between the coding team and the trial representative was calculated as percentage agreement (85.3%). When a member of the study team was also a trial investigator, an independent trial representative completed the verification (*k* = 1).

### 2.6 Synthesis Methods

Intervention arms were the unit of analysis, as trials often included more than one weight management intervention. For this study, active control arms were also classified as intervention arms and included in coding. Descriptive statistics were used to describe the frequency (count and percent) of each discrete intervention component across all interventions, with analyses performed in SPSS Statistics, version 28.0 (IBM Inc). Heat maps were prepared to display the proportion of possible intervention strategies used per intervention arm. Network diagrams were prepared in Flourish (Kiln Enterprises Ltd. http://flourish.studio) to display the frequency of intervention strategy clusters and relationships between clusters used across intervention arms. Given the changes made to coding during the validation process, only validated intervention arms were included in primary analyses. Sensitivity analyses were conducted with all eligible trials, using data coded from published materials only (i.e., prior to updating via the validation process) (Supporting Information S4). Coding datasets are available upon request to the corresponding author.

## 3 Results

### 3.1 Study Selection and Sample Characteristics

**FIGURE 1:**
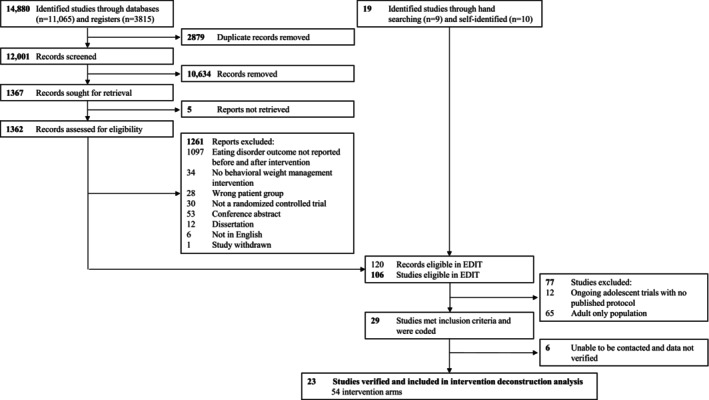
PRISMA flow diagram.

Of the 14 880 articles identified in the search, 29 met inclusion criteria and were coded [29, 30, 31, 32, 33, 34, 35, 36, 37, 38, 39, 40, 41, 42, 43, 44, 45, 46, 47, 48, 49, 50, 51, 52, 53, 54, 55, 56, 57, 58, 59, 60, 61, 62, 63, 64, 65, 66, 67]. Twenty‐three trials [29, 30, 31, 32, 33, 34, 35, 36, 37, 38, 39, 40, 41, 42, 43, 44, 45, 46, 47, 48, 49, 50, 51, 52, 53, 54, 55, 56, 57, 58, 59, 60, 61, 62, 63, 64, 65, 66, 67, 68, 69, 70, 71, 72, 73, 74, 75, 76] with 54 intervention arms (referred to hereafter as interventions[k]) were verified with trialists and included in this study (Figure 1). Included trials were published from 2000 to 2020 and are listed in Table 1. Trials were conducted in the United States (*n* = 14), Belgium (*n* = 3), Australia (*n* = 2), Brazil (*n* = 2), Norway (*n* = 1) and England (*n* = 1).

**TABLE 1:** Table of characteristics for verified and included trials.

*Study details* [first author, year of publication; country; setting (hospital outpatient/inpatient, university, community etc.)]	*Participant characteristics* [sample size (*n*) %female (F), mean age (SD) in years]	*Intervention details* [intervention duration, duration of follow‐up (FU) (from post‐intervention) delivery, intensity (group/individual/with support person, frequency of contact)]
Bean et al. 2022 [29]; Raynor et al. 2021 [30]; USA; primary care	*n* = 82 63.4% (F), 13.7 (1.2) years	4 months, FU 3 months Individual with support person and group, weekly
Bonham et al. 2017 [31]; Dordevic et al. 2015 [32]; Australia; commercial provider	*n* = 74 74.3% (F), 15.3 (1.3) years	*JenMe*: 12 weeks, FU 24 weeks^a^ Individual with support person, weekly
Boutelle et al. 2011 [33]; USA; University	*n* = 36 58% (F), 0.3 (1.3) years	8 weeks, FU 12 months Individual with support person and group, weekly
Boutelle et al. 2017 [34]; Eichen et al. 2019 [33]; Boutelle et al. 2015 [36]; USA; University	*n* = 150 66.7% (F), 10.4 (1.3) years	6 months, FU 18 months Both arms used same intensity, that is weekly, but differed by delivery *FBT*: individual with support person, family approach and group *PBT*: individual, family approach and group
Braet and Van Winckel 2000 [37]; Belgium; Hospital outpatient	*n* = 136 68.4% (F), 11 (2.5) years	1 year, FU 3.6 years All arms used stage approach (e.g., weekly > monthly) delivery, but differed by intensity *Group CBM*: individual with support person and group *Individual CBM*: individual with support person *Summer camp CMB*: individual with support person and group
Braet et al. 2004 [38]; Belgium; Hospital inpatient	*n* = 150 63.3% (F) Data for *n* = 122 completers: 12.7 (2.3) years	10 months, FU 4 months Individual with support person and group, less than weekly
Croker et al. 2012 [39]; England; Hospital outpatient	*n* = 72 69.4 (F), 10.3 (1.6) years	*FBBT*: 6 months, FU 6 months^a^ Family approach and group, stage approach (e.g., weekly > monthly)
de Lira et al. 2017 [40]; Tenório et al. 2018 [41]; Fidelix et al. 2019 [42]; Brazil; Hospital outpatient and University	*n* = 107 58.9% (F), 15 (1) years	*HIT & LIT*:^a^ 12 weeks, no FU Individual and group, less than weekly
Doyle et al. 2008 [43]; USA; Virtual and University	*n* = 83, data for *n* = 80^b^: 62.5% (F), 14.5 (1.7) years	16 weeks, FU 4 months Both arms differed by delivery and intensity *SB2*: individual with support person and group, weekly *Usual care*: individual with support person, intensity could not be calculated
Epstein et al. 2001 [44]; USA; Community	*n* = 67 Data for *n* = 47 completers: 46.8% (F), 10.3 (1.1) years	6 months, FU 1.5 years Individual with support person and group, stage approach (e.g., weekly > monthly)
Jelalian et al. 2006 [45]; USA; Hospital outpatient	*n* = 89 Data for *n* = 76^c^: 71% (F), 14.51 (0.9) years	*CBT + EXER* & *CBT + PEAT*: 32 weeks, no FU *Standard care*: 16 weeks, no FU All arms used same intensity, that is stage approach (e.g., weekly > monthly), but differed by delivery *CBT + EXER* & *CBT + PEAT*: individual with support person and group *Standard care*: individual
Jelalian et al. 2020 [46]; Darling et al. 2021 [47]; USA; Community	*n* = 66 60.6% (F), 14.7 (1.6) years	10 months, no FU Individual with support person and group, stage approach (e.g., weekly > monthly)
Lister et al. 2024 [48]; Jebeile et al. 2024 [49]; Lister et al. 2020 [50]; Australia; Hospital outpatient & virtual	*n* = 141 49.6% (F), Median 14.8 (IQR 13.8–15.7) years	52 weeks, FU 52 weeks Individual with support person, stage approach (e.g., weekly > monthly)
Lofrano‐Prado et al. 2022 [51]; Brazil; School	*n* = 74 54.1% (F), 15 (1) years	24 weeks, no FU Group, stage approach (e.g., weekly > monthly)
Mehlenbeck et al. 2009 [52]; Jelalian et al. 2010 [53]; USA; Hospital outpatient	*n* = 118 68% (F), 14.3 (1.0) years	52 weeks, FU 1 year Individual with support person and group, stage approach (e.g., weekly > monthly)
Newsome et al. 2023 [54]; USA; Virtual	*n* = 40 100% (F), 15.8 (1.5) years	6 months, no FU Individual with support person, stage approach (e.g., weekly > monthly)
Shomaker et al. 2017 [55]; USA; Hospital outpatient	*n* = 29 62.1% (F), *FB‐IPT* 11.7 (1.6) years, *FB‐HE* 11.0 (1.9) years	12 weeks, FU 9 months Individual with support person, weekly
Skjåkødegård et al. 2022 [56]; Skjåkødegård et al. 2016 [57]; Norway; Hospital outpatient	*n* = 114 *FBSFT* 61.0% (F), 12.6 (3.3) years *TAU* 56.4% (F), 12.6 (2.8) years	6 months, FU 1.5 years Both arms used same delivery, that is individual with support person and family approach, but differed in intensity *FBSFT*: every 2–3 weeks *TAU*: stage approach (e.g., weekly > monthly)
Vermeiren et al. 2021 [58]; Naets et al. 2018 [59]; Tanghe et al. 2020 [60]; Belgium; Hospital inpatient and outpatient	*n* = 259 *Inpatient* 58.3% (F), 14.3 (2.2) years *Outpatient* 54.8% (F), 11.9 (2.1) years	12 weeks, FU 6 months Both arms differed by delivery and intensity *Inpatient*: individual with support person and group, less than weekly *Outpatient*: individual with support person, every 2–3 weeks
Vidmar et al. 2021 [61]; Vidmar et al. 2020 [62]; USA; Hospital outpatient and virtual	*n* = 50 72.0% (F), 16.4 (1.2) years	12 weeks, no FU Individual, weekly
Vidmar et al. 2023 [63]; Vidmar et al. 2019 [64]; USA; Hospital outpatient and virtual	*n* = 161 65% (F), 16 (2.5) years	6 months, FU 12 months All arms differed by delivery and intensity *App*: individual, less than weekly *App + Coach*: individual, stage approach (e.g., weekly > monthly) *Clinic*: individual with support person, monthly
Wilfley et al. 2017 [65]; USA; University	*n* = 172 61.6% (F), 9.4 (1.3) years	4 months, FU 8 months Individual with support person, family and group, weekly
Wilfley et al. 2007 [66]; Goldschmidt et al. 2014 [67]; USA; University	*n* = 150 69.3% (F), 9.9 (1.3) years	*BSM & SFM*: 9 months, FU 20 months Usual care: 5 months, FU 24 months Individual with support person, weekly

Abbreviations: BSM, behavioural skills maintenance; CBT + EXER, cognitive‐behavioural treatment with aerobic exercise; CBT + PEAT, cognitive‐behavioural treatment with peer‐enhanced adventure therapy; CMB, cognitive‐behaviour modification; FBBT, family‐based behavioural treatment; FBSFT, family‐based behavioural social facilitation treatment; FBT, family‐based behavioural treatment; FU, follow‐up; HIT, high‐intensity training; LIT, low‐intensity training; PBT, parent‐only variant of FBT; SB2, student bodies 2; SFM, social facilitation maintenance; TAU, treatment as usual.

^a^ The study included a no‐intervention waitlist control arm which was not included in this analysis.

^b^ Three participants were excluded post randomisation.

^c^ The standard care arm was discontinued.

### 3.2 Delivery Features

The frequency of delivery features across interventions is reported in Table 2. Most interventions aimed for a combination of weight loss and maintenance (54%) and were informed by a psychological theory or framework, most commonly Cognitive Behavioural Theory (43%). Interventions commonly included a support person with the adolescent (70%), provision of information sheets/booklets (93%), materials to assist with dietary monitoring, (56%) and face‐to‐face delivery (93%). Interventions were delivered by trained health professionals (80%), commonly including a psychologist or counsellor (76%) or dietitian or nutritionist (65%). A range of outcomes was reported as part of interventions with most weighing adolescents (96%) and measuring psychosocial or mental health (83%).

**TABLE 2:** Delivery features intervention arm summary.

Cluster	Components	Frequency in intervention arms (%) *k* = 54
Why—theory	Weight maintenance	5 (9)
	Weight loss	20 (37)
	Weight loss and maintenance	29 (54)
Psychological theory or framework underpinning intervention	Cognitive behavioural therapy (CBT)	23 (43)
	Enhanced cognitive behavioural therapy (CBT‐E)	0 (0)
	Acceptance and commitment therapy (ACT)	1 (2)
	Dialectical behaviour therapy (DBT)	0 (0)
	Family‐based therapy (FBT)	13 (24)
	Interpersonal therapy (IPT) for binge eating	2 (4)
	Trauma‐informed care	0 (0)
	Compassion focussed therapy	0 (0)
	Motivational interviewing	11 (20)
	Other General theory (not based on psychological theory)	24 (44)
Target population/recipient of the intervention—AGE GROUP	Adolescent	54 (100)
	Adult	10 (19)
Target population/recipient of the intervention—WEIGHT category	Overweight	39 (72)
	Obesity	54 (100)
	Severe obesity	51 (94)
Target population/recipient of the intervention—individual versus with support person/s	Individual	12 (22)
	Individual with support person	38 (70)
	Family or household‐based treatment approach	13 (24)
What—materials	Information sheets/booklets	50 (93)
	Food	8 (15)
	Diet monitoring materials	30 (56)
	Supplements	4 (7)
	Meal replacement products	3 (6)
	Sports equipment	11 (20)
	Fitness/activity tracker	12 (22)
	Body scales	12 (22)
	Food scales	6 (11)
	Mobile app	6 (11)
	Website access (online resources)	5 (9)
	Social media	2 (4)
	Other materials	7 (13)
What—procedures	Nutrition education	51 (94)
	Energy prescription or target	27 (50)
	Physical activity education	47 (87)
	Exercise classes	16 (30)
	Psychological component	44 (81)
What—outcome measures	Weight/adiposity	54 (100)
	Physical health outcomes	20 (37)
	Psychosocial/mental health outcomes:	45 (83)
	Eating behaviour outcomes	21 (39)
	Individual weighing at visits	52 (96)
	Blind weighing at visits	5 (9)
	Group weighing	0 (0)
	Communication about the ability to decline weight or opt out of weighing during visits	8 (15)
Who provided—intervention delivered by (personnel, training, qualifications)	Dietitian/nutritionist	35 (65)
	Nurse	5 (9)
	Exercise physiologist/physiotherapist/personal trainer/other exercise professional	21 (39)
	Psychologist/counsellor	41 (76)
	Physician—paediatrician, GP, endocrinologist	16 (30)
	Pharmacist	0 (0)
	Researcher/non‐health professional	17 (31)
	Self‐delivered	1 (2)
	Other	6 (11)
Training for interventionist	Training for the interventionist	43 (80)
How—delivery mode	Face‐to‐face	50 (93)
	Computer/Web‐based/Online	12 (22)
	Call (telephone, video)/SMS	9 (17)
	Printed material	34 (63)
	Individual	44 (81)
	Group	38 (70)
	Peer support	5 (9)
Where—intervention setting	Hospital outpatient	22 (41)
	Hospital inpatient	5 (9)
	University/research centre	13 (24)
	Primary care	2 (4)
	Community	2 (4)
	School	2 (4)
	Household residence	0 (0)
	Virtual	12 (22)
	Commercial provider centres	1 (2)
	Workplace	0 (0)
Post‐intervention support	Referral to other services	6 (11)
	Additional information provided	4 (7)
	Other	1 (2)
Tailoring	Was there an element of tailoring in the intervention?	33 (61)
Modifications	Was the intervention modified?	7 (13)
Fidelity	Were any intervention fidelity procedures (actual or planned) described?	45 (83)

Overall intervention duration was available for 45 interventions and ranged from 8 to 52 weeks (median 26). Total number of contacts ranged from 1 to 149 (median 20). Total duration of contact ranged from 5 to 63 h (median 22.5 h). The most common approach to frequency of contacts was a staged approach, where visits began weekly and then spread to monthly over the course of the intervention (41%) or having regular weekly visits (35%).

### 3.3 Intervention Strategies

**FIGURE 2:**
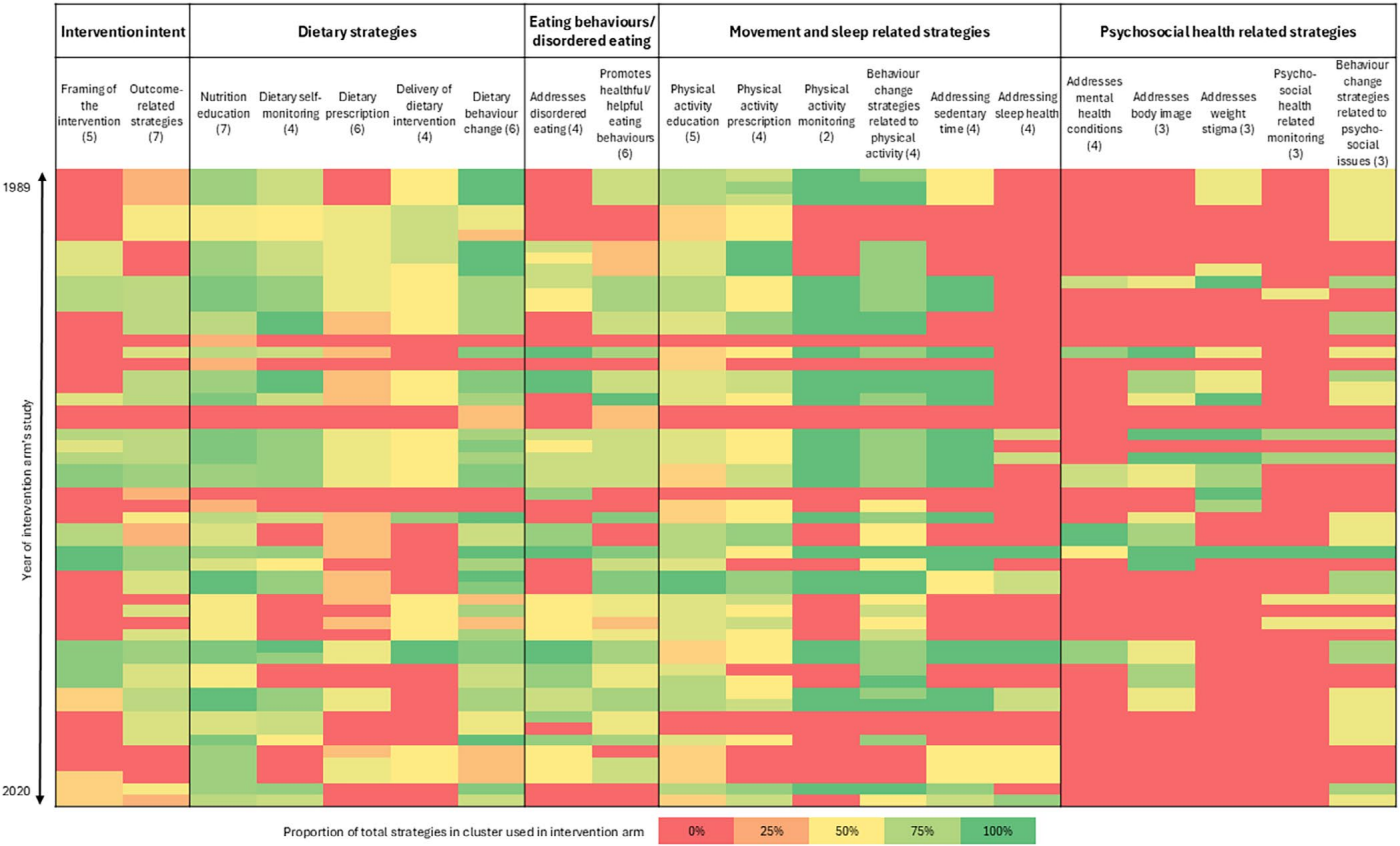
Frequency of intervention strategies across and within intervention arms. Each row represents an intervention arm arranged by year of the corresponding trial, starting from the oldest trial at the top down to the most recent trial (based on recruitment date, or publication date if unavailable). Each column represents clusters of intervention strategies within five broad categories; each number in brackets reports the number of strategies within that cluster. The colors of each square depict the proportion of total intervention strategies within that strategy cluster that are used in the intervention arm.

Interventions had a mean (SD) of 30 (16.1) intervention strategies. Across the five categories of intervention strategies, interventions commonly included strategies within the *Dietary Strategies* category (98%), *Eating Behaviour and Disordered Eating* category (85%) and the *Movement and Sleep Related Strategies* category (89%), with fewer interventions including strategies from the *Psychosocial Health Related Strategies* category (76%) (Supporting Information S5). Across all clusters, trials were most likely to have included strategies from the *Nutrition Education* cluster (*k* = 51, 94%), *Dietary Behaviour Change* cluster (*k* = 50, 93%) and the *Physical Activity Education* cluster (*k* = 48, 89%). Few interventions included strategies from the *Psychosocial Health Related Monitoring* cluster (*k* = 6, 11%). The frequency of intervention strategies within each intervention arm and grouped by intervention cluster and category is shown in the heatmap in Figure 2 and Supporting Information S5. The most common intervention strategies within each category and cluster are described below.

#### 3.3.1 Intervention Intent Category (Two Clusters)

The most common strategy within the *Framing of the Intervention (communications)* cluster was providing education that health behaviours are linked to health outcomes (*k* = 22, 41%), with few interventions providing education that obesity is a disease (*k* = 8, 15%). The most common strategies in the *Outcome Related Strategies* cluster were the use of weight‐focused goals (*k* = 33, 61%) and providing feedback on weight (*k* = 33, 61%), with few providing feedback on changes in metabolic health outcomes (*k* = 7, 13%).

#### 3.3.2 Dietary Strategies Category (Five Clusters)

The most common strategies in the *Nutrition Education* cluster were to provide healthy eating (i.e., serve size and food group recommendations; *k* = 48, 89%) and portion size advice (*k* = 40, 74%). Within the *Dietary Self‐monitoring* cluster, the most common strategies were using food‐based dietary self‐monitoring (*k* = 32, 59%) and providing feedback on this monitoring (*k* = 30, 56%). A hypocaloric diet (*k* = 26, 48%) was the most frequently used *Dietary Prescription* strategy, and flexible meal plans (*k* = 30, 56%) were the most common strategy used to support the *Delivery of the Dietary Intervention*. Most interventions used strategies in the *Dietary Behaviour Change* cluster, with problem solving (*k* = 43, 80%), goal setting (*k* = 41, 76%), and addressing the environment (*k* = 36, 67%) most frequently used. Few trials used very low energy diets or meal replacement products (*k* = 2, 4%) or promoted the *ad libitum* intake of ‘free foods’ (*k* = 3, 6%).

#### 3.3.3 Eating Behaviours/Disordered Eating Category (Two Clusters)

The most common strategy in the cluster on *Addressing Disordered Eating*, was identifying disordered eating behaviours (*k* = 29, 54%), with relatively few providing education on the risk of eating disorders (*k* = 10, 19%). Within the cluster *Promote Healthful/Helpful Eating Behaviours*, half of the trials used at least one strategy, with promoting mealtime routines (*k* = 30, 56%) and promoting mealtime support (*k* = 28, 52%) being the most common. Only two (4%) interventions encouraged intuitive eating principles or practice.

#### 3.3.4 Movement and Sleep Strategies Category (Six Clusters)

Within the *Physical Activity Education* cluster, most interventions provided education to increase physical activity (*k* = 48, 89%) and promoted joyful movement and activity (*k* = 24, 44%), with few providing cultural adaptations to physical activity (*k* = 2, 4%). Most interventions provided a flexible exercise plan (k = 37, 69%) within the *Physical Activity Prescription* cluster and encouraged self‐monitoring of activity, with feedback provided by about half of the interventions (*k* = 26, 48%) within the *Physical Activity Monitoring* cluster. *Behaviour Change Strategies Related to Physical Activity* commonly included encouraging activity‐focused goals (*k* = 38, 70%) and problem‐solving barriers to physical activity (*k* = 33, 61%). As part of *Addressing Sedentary Time*, trials provided education to reduce/limit sedentary time (*k* = 29, 54%), and few included strategies for *Addressing Sleep Health*, with 13 interventions (24%) providing education on sleep health.

#### 3.3.5 Psychosocial Health Strategies Category (Five Clusters)

Overall, few interventions included strategies within this category. Within the cluster *Addresses Mental Health Conditions*, seven interventions (13%) had protocolized the identification of mental health conditions and referring to psychological support. However, it was noted during verification meetings that trials regularly did this as part of routine clinical care and it was not protocolized as a specific intervention component. Some interventions addressed body image concerns (*k* = 17, 31%) and promoted body compassion, acceptance and/or positivity (*k* = 12, 22%) within the *Addresses Body Image* cluster. Within the *Addresses Weight Stigma* cluster, some interventions provided education and/or strategies to increase resilience to weight stigma, bullying, teasing (*k* = 13, 24%) or education to support people about weight stigma and teasing (*k* = 10, 19%). Where *Psychosocial Health Related Monitoring* strategies were used, this included self‐monitoring of thoughts, feelings, mood (*k* = 6, 11%). *Behaviour Change Strategies Related to Psychosocial Health* strategies were most commonly used within this category, with inclusion of peer/social support strategies (*k* = 21, 39%) or increased skills to manage psychosocial health (*k* = 17, 31%) being the most common.

### 3.4 Combinations of Intervention Strategies Clusters

**FIGURE 3:**
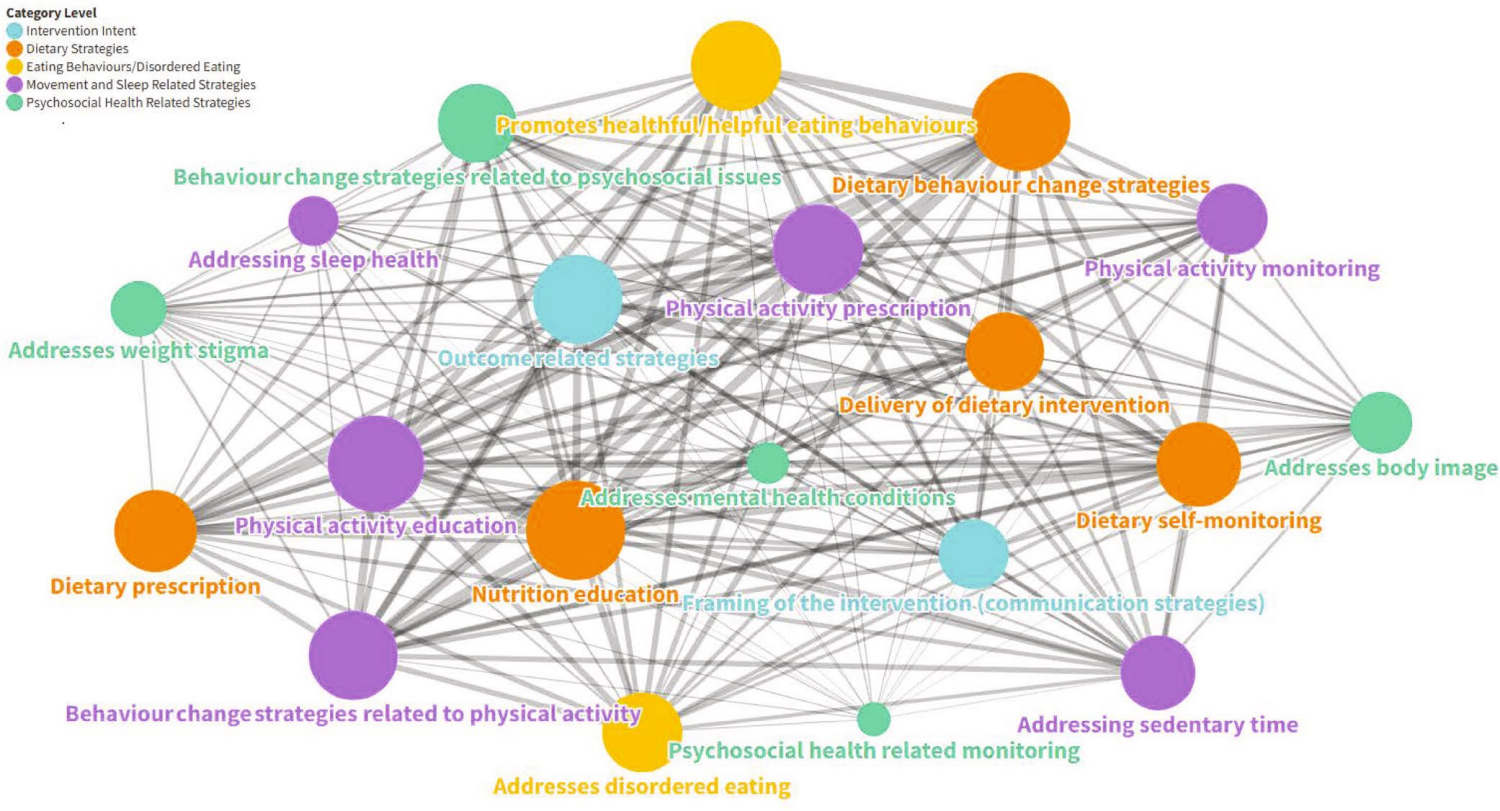
Frequency of individual and combinations of intervention strategy clusters across all interventions. Circles represent clusters of intervention strategy clusters with circle colours corresponding to category levels. Frequency of intervention strategy clusters is reflected by circle sizes with larger circles representing more common strategy clusters. Combinations and frequency strategy clusters are reflected by line width and connections, with thicker lines representing more common combinations.

Figure 3 shows clusters of strategies linked together for all interventions. Clusters with more strategies are represented by larger circles (e.g., see nutrition education), whereas clusters with fewer strategies are represented by smaller circles (e.g., see psychosocial health‐related monitoring). The width of the connecting lines represents more interventions with both clusters: for example, nutrition education and dietary self‐monitoring frequently co‐occurred, while psychosocial health‐related monitoring and addressing sedentary time rarely co‐occurred. The number of connections in the figure highlights the diversity in the combination of intervention clusters identified in included interventions.

## 4 Discussion

This review provides an in‐depth analysis of components of adolescent behavioural weight management interventions that measure eating disorder outcomes using a project‐specific framework. The most common intervention strategies used were Nutrition Education, Dietary Behaviour Change and Physical Activity Education, with few interventions including Psychosocial Health‐related strategies. The range of delivery features and intervention strategies included within each intervention arm highlights the complexity of weight management interventions eligible for the EDIT Collaboration. We found that interventions varied greatly in both the delivery features and intervention strategies. The intervention strategies addressed five key categories related to behavioural weight management. Most interventions included behaviour change strategies in all categories; however, our detailed mapping demonstrates the number of strategies within each category varied across interventions. For example, many interventions described a psychological theory or framework that underpinned the intervention *delivery*, yet—as coded in intervention strategies—few studies targeted psychosocial health behaviours, such as addressing self‐esteem or body image concerns. Thus, the intervention deconstruction methodology explored a greater depth of intervention components, furthering our understanding of the context and complexity of adolescent weight management interventions. This review demonstrates adolescent weight management clinical trials include many overlapping components, which has implications when synthesising evidence to inform personalised care. Further research is needed to understand how components are implemented in clinical practice. Mapping exposure to intervention components for an individual is needed to understand how weight management impacts risks and benefits of an intervention.

The interventions included in this review involved measurement of eating disorder risk before and after the intervention, and therefore findings may not be generalisable to weight management interventions not measuring eating disorder risk. It is possible that included interventions were designed by clinicians and researchers more cognisant of the risk of eating disorders. Understanding the influence of measuring and thus acknowledging the risk of eating disorders in behavioural weight management is beyond the scope of this review. However, our framework can be applied to other weight management research studies and could provide insight into differing intervention designs and outcomes. Although 65% of interventions included a ‘dietary prescription’, which in the context of weight management, is perceived to increase eating disorder risk [26], many of these interventions also included strategies to promote healthful/helpful eating behaviours. It is unclear how such combinations of interventions may influence eating disorder risk. Research is needed to better understand contributions to eating disorder risk when these strategies are combined during interventions.

The depth of our intervention coding approach limits comparisons that can be made with the broader literature, as few studies have examined detailed delivery features and intervention strategies. Past reviews in paediatric weight management more broadly have found components such as self‐monitoring [77] have small, but significant effect sizes for improving weight status. A 2013 review examining the efficacy and effectiveness of behaviour change techniques [11] highlighted key issues we address in this study. Firstly, earlier taxonomies were not comprehensive enough to capture all intervention strategies reported in childhood and adolescent obesity interventions. Previous taxonomies focus on behaviour change techniques or intervention delivery. However, intervention content is also important. Thus our study implemented a project‐specific framework [22], capturing strategies identified in a broad consultation process [26], that were likely to influence efficacy/effectiveness and eating disorder risk in behavioural weight management. Secondly, the synthesis of intervention strategies in this review and resultant network maps shows a complex interaction of several behaviour change approaches within each intervention arm. This highlights the complexity of behavioural interventions and is supported by earlier findings that the most successful interventions for weight loss include combinations of behaviour change techniques [11].

In traditional methods of evidence synthesis of treatment interventions, sub‐groups are often formed based on key features of an intervention. For example, in our 2019 meta‐analysis [6] examining eating disorder outcomes, sub‐groups comparing nutrition education alone to energy prescription and physical activity education to exercise classes were used to explore heterogeneity between interventions. Similarly, in community studies exploring prospective risk factors for eating disorders, dietary behaviours are often broadly classified as healthy or unhealthy [13]. In both instances, little consideration is given to the broader context of these behaviours, and concurrent support strategies that may be in place. Moving from broad categorizations to detailed coding, as used in this review, may be needed to understand ‘active ingredients’ influencing both treatment outcomes and safety and to inform personalised care. Further, personalised care should also consider the characteristics of the individual that may also interact with interventions and their components. Thus, future research should focus on identifying opportunities for examining the effects of different intervention strategies and technique combinations on safety and effectiveness outcomes for different populations or different individuals. The EDIT Collaboration will examine how intervention components influence eating disorder risk [17].

The coding framework for this study was developed for use by the EDIT Collaboration to describe a broad range of behavioural interventions. However, this framework could be adapted and applied to other treatment interventions and studies in other populations to inform standards of care. A helpful next step would be to apply our framework to other interventions (e.g., RCTs without eating disorder measures, behavioural interventions supporting pharmacotherapy) delivered in adolescent obesity populations to facilitate comparison in safety and effectiveness. Future research should also consider how detailed coding frameworks such as this can be incorporated into reporting of clinical trials and traditional methods of evidence synthesis to explain and account for heterogeneity of treatment effects in this complex area. A process evaluation in future research will provide insights for further adapting the intervention deconstruction methodology. For example, it might be helpful to develop definitions to allow sub‐grouping interventions based on the complexity of the interventions rather than the presence or absence of a particular strategy or behaviour change technique.

Our study had several strengths. Firstly, we used an extensive search including searching clinical trial registries to identify all eligible interventions, then applied a project‐specific systematic coding method which included validating codes with trial investigators. This allowed for a comprehensive description of intervention components, with the coding framework developed with a broad range of stakeholders. Limitations included coding conducted by a single coder using published descriptions that often underreport intervention components [23], which was somewhat mitigated by the validation process with the trial investigator. Our eligibility criteria excluded pharmacotherapy and surgical interventions, which may in part explain why included interventions tended to be older (59% were published > 10 years ago). Verification of codes was reliant on trialist self‐report. We did not code the dose/intensity of components, only the presence or absence and thus may have missed a ‘threshold’ effect for a particular intervention component. An important limitation is that interventions included in this review had to assess eating disorder risk before and after the intervention, and therefore likely do not represent all weight management interventions. The potential for publication bias, whereby only favourable eating disorder outcomes are published, was addressed by searching clinical trial registries.

## 5 Conclusion

Adolescent weight management interventions are complex and vary in delivery features and intervention strategies. Understanding interventions is a critical first step to designing weight management interventions to reduce eating disorder risk.

## Conflicts of Interest

N.B.L., R.K., I.R.J., S.P., H.M., A.L.S, K.E.H., C.B., S.P.G., S.M., D.N., S.J.P., K.S., K.C., G.D., A.M.G., F.Q., M.R., J.Y., M.K.B., M.P.B., K.N.B., K.E.D., D.M.E., L.H.E., A.B.G., E.J., M.C.L.‐P., T.N., W.L.P., R.I.S., M.T.‐K., A.V.E., B.J.J., H.J. declare no conflicts of interest. A.L.A. is on the Scientific Advisory Board for WW and chairs a Trial Steering Committee for Menwell Ltd. L.A.B. is immediate Past‐President (2022–2024) of the World Obesity Federation, an honorary, unpaid position. She has received honoraria for speaking in forums organised by Novo Nordisk (NN) in relation to management of adolescent obesity (funds directed to research cost centre). L.A.B. is the Australian lead of the study—ACTION Teens: sponsored by NN (multi‐country on‐line study of attitudes towards and perceptions of obesity held by adolescents living with obesity, their parents and health care professionals). L.A.B. has received honoraria (funds directed to hospital research cost centre) for being a member of the Lilly Weight Management Advisory Board. A.J.H. has received payment for advice given to Slimming World (UK). M.K.P. has received travel and consulting fees for speaking and advisory boards from Novo Nordisk, Eli Lilly, Johnson and Johnson Medical Pty Ltd, Takeda Pharmaceuticals and iNova Pharmaceuticals. He was on the guideline development committee for the National Eating Disorder Collaboration ‘Management of eating disorders for people with higher weight: clinical practice guideline (2022)’ and is Vice President of the Australia and New Zealand Obesity Society (ANZOS). A.S. owns 50% of the shares in Zuman International Pty Ltd, which receives royalties and other payments for educational resources and services in adult weight management and research methodology. F.A.N.H. reports serving on the Obesity Society's Finance Committee, consulting fees from WW, an honorarium for speaking from the American Academy of Pediatrics, institutional support to travel and membership on the Obesity Action Coalition's Membership and Access to Care Committees, outside the scope of the submitted work. R.A.J. is an employee and shareholder of WeightWatchers International (WW). T.K.K. has received professional fees from Novo Nordisk, Nutrisystem, Emerald Lake Safety, Roman Health Ventures and Boehringer Ingelheim. M.I.C. is an employee and shareholder of WW International Inc. A.L.D. has an honorary, unpaid position at Nutrition Society of Australia as Honorary Secretary and Director of the Board from 2024. H.F.S. previously received salary from Novo Nordisk unrelated to the present work. Y.S.D. received a one‐time payment from NovoNordisk for creating information materials about eating disorders for health care professionals. Y.S.D. is part of the steering group of the E‐BATTLE Obesity trial a placebo‐controlled RCT testing semaglutide in the treatment of severe obesity in adolescents (starting in 2025). Medications and placebo‐medications are provided by Novo Nordisk but not funding for the study. A.P.V. has received consulting fees from Rhythm Pharmaceuticals and Soleno Pharmaceuticals. J.A.Y. reports unrelated grant funds to NICHD supporting his clinical research from Soleno Therapeutics, Rhythm Pharmaceuticals and Hikma Pharmaceuticals and materials from Versanis Bio to support mouse studies.

## Supporting information


**Data S1:** ijpo70074‐sup‐0001‐Supinfo1.pdf.

## Data Availability

The data that support the findings of this study are available from the corresponding author upon reasonable request.
