# Supplementary material for: Components of Adolescent Behavioural Interventions With Eating Disorder Outcomes: Systematic Review With Intervention Mapping
Source: Pediatr Obes. 2025 Dec 10;21(1):e70074. doi: 10.1111/ijpo.70074 (PMC12690354; doi:10.1111/ijpo.70074)
Supplement: Supplementary file 1 — Data S1: ijpo70074‐sup‐0001‐Supinfo1.pdf. [file IJPO-21-e70074-s001.pdf]

Supplementary File S1: Changes to the Protocol from Original Published Protocol.

Number	Description
1	Description of training for the interventionist added (10.2 Describe)
2	Extra option of ‘weekly’ added to intervention dose: intensity (13.4)
3	Post intervention support ‘Other’ added (14.3 Other)
4	Merged fidelity codes ‘planned fidelity measures’ and ‘actual fidelity measures’ together in one code (17.1 ‘Were any intervention fidelity procedures (actual or planned) described?’)
5	Addition of one intervention strategy, 18.6 ‘Language’- weight focused language or health focused language or a combination of weight and health focused language

Supplementary File S2: Search strategies.

Database: Ovid MEDLINE(R) ALL
Search Strategy:
----
1 exp Obesity/
2 exp Overweight/
3 obes*.tw.
4 overweight.tw.
5 1 or 2 or 3 or 4
6 weight loss/
7 exp diet therapy/
8 exp bariatrics/
9 exp exercise/
10 anti-obesity agents/ or appetite depressants/
11 (diet* adj2 therap*).tw.
12 bariatric*.tw.
13 (low adj3 (energy or calor*) adj4 diet).tw.
14 ((pharma* or diet* or obes* or lifestyle or behavio*) adj3 (interven* or treat* or therap*)).tw.
15 ((calori* or diet*) adj3 (reduc* or restrict*)).tw.
16 (weight adj3 (manag* or los*)).tw.
17 (exercis* or physical activit*).tw.
18 HAES.mp.
19 health at every size.mp.
20 (weight adj2 neutral).mp.
21 nondiet.mp.
22 (non adj2 diet).mp.
23 (intuitive adj2 eat*).mp.
24 mindful*.tw. (9839)
25 6 or 7 or 8 or 9 or 10 or 11 or 12 or 13 or 14 or 15 or 16 or 17 or 18 or 19 or 20 or 21 or 22 or 23 or 24
26 Body Image/
27 (body adj3 (accept* or dissatisf* or image or satisf* or appreciat* or esteem)).tw.
28 "feeding and eating disorders"/ or anorexia nervosa/ or binge-eating disorder/ or bulimia nervosa/ or "feeding and eating disorders of childhood"/
29 (bulimi* adj3 symptom*).tw.
30 (disorder* adj3 eat*).tw.
31 (emotion* adj3 eat*).tw.
32 (diet* adj3 restr*).tw.
33 (binge adj3 eat*).tw.
34 extreme weight loss.tw.
35 loss of control.tw.
36 drive for thinness.tw.
37 ((weight or shape or eat*) adj3 concern).tw.
38 (eat* adj2 behavi*).tw.

39 26 or 27 or 28 or 29 or 30 or 31 or 32 or 33 or 34 or 35 or 36 or 37 or 38
40 randomized controlled trial/
41 (randomi?ed controlled trial* or RCT* or (controlled adj3 trial)).mp.
42 randomi?ed.ti.
43 clinical trials as topic.sh.
44 randomly.ab.
45 trial.mp.
46 clinical trial.mp.
47 40 or 41 or 42 or 43 or 44 or 45 or 46
48 5 and 25 and 39 and 47

****

RCT related key words
"clinical trials" OR "clinical trials as a topic" OR "randomized controlled trial" OR "Randomized Controlled Trials as Topic" OR "controlled clinical trial" OR "Controlled Clinical Trials as Topic" OR "random allocation" OR "randomly allocated" OR "allocated randomly" OR "Double-Blind Method" OR "Single-Blind Method" OR "Cross-Over Studies" OR "Placebos" OR "cross-over trial" OR "single blind" OR "double blind" OR "factorial design" OR "factorial trial" OR clinical trial* OR trial* OR rct* OR random* OR blind*

SCOPUS

Obes* OR Overweight*
AND

“Weight loss” OR “Diet* OR bariatric* OR Exercis* OR “anti-obesity agent*” OR HAES OR “health at every size” OR “Weight neutral” OR “Intuitive eat*” OR Mindful*

((pharma* or diet* or obes* or lifestyle or behavio*) W/4 (interven* or treat* or therap*))

AND
“Body image*” OR “Eating disorder*” OR anorexia OR “binge-eating disorder*” OR bulimi* OR
“Emotion* eat*” OR Diet* restr*” OR “Binge eat*” OR "extreme weight loss*" OR “loss of Control” OR “Drive for thinness”
((weight or shape or eat*) W/3 concern*)

Scopus complete query

(TITLE-ABS-KEY ("clinical trials" OR "clinical trials as a topic" OR "randomized controlled trial" OR "Randomized Controlled Trials as Topic" OR "controlled clinical trial" OR "Controlled Clinical Trials as Topic" OR "Clinical trial*" OR trial* OR rct OR random*)) AND ((TITLE-ABS-KEY (obes* OR overweight*)) AND ((TITLE-ABS-KEY ("Weight loss" OR diet* OR bariatric* OR exercis* OR "anti-

obesity agent*" OR haes OR "health at every size" OR "Weight neutral" OR "Intuitive eat*" OR mindful*)) OR (TITLE-ABS-KEY (((pharma* OR diet* OR obes* OR lifestyle OR behavio*) W/4 (interven* OR treat* OR therap*)))))) AND ((TITLE-ABS-KEY (((weight OR shape OR eat*) W/3 concern*))) OR ((TITLE-ABS-KEY ("Body image*" OR "Eating disorder*" OR anorexia OR "binge eating disorder*" OR bulimi* OR "Emotion* eat*" OR "Diet* restr*" OR "Binge eat*" OR "extreme weight loss*" OR "loss of Control" OR "Drive for thinness")) OR (TITLE-ABS-KEY (((weight OR shape OR eat*) W/3 concern*))))))

Database: Embase
Search Strategy:
----
1 obesity/
2 obes*.tw.
3 overweight.tw.
4 1 or 2 or 3
5 weight reduction/
6 diet therapy/ or diet restriction/ or low calory diet/ or low fat diet/
7 bariatric surgery/ or gastric banding/ or sleeve gastrectomy/
8 exercise/
9 antiobesity agent/
10 (diet* adj2 therap*).tw.
11 bariatric*.tw.
12 (low adj4 (energy or calor*) adj4 diet).tw.
13 ((pharma* or diet* or obes* or lifestyle or behavio*) adj3 (interven* or treat* or therap*)).tw.
14 ((calori* or diet*) adj3 (reduc* or restrict*)).tw.
15 (weight adj3 (manag* or los*)).tw.
16 (exercis* or physical activit*).tw.
17 HAES.mp.
18 health at every size.mp.
19 (weight adj2 neutral).mp.
20 nondiet.mp.
21 (non adj2 diet).mp.
22 (intuitive adj2 eat*).mp.
23 mindful*.tw.
24 5 or 6 or 7 or 8 or 9 or 10 or 11 or 12 or 13 or 14 or 15 or 16 or 17 or 18 or 19 or 20 or 21 or 22 or 23
25 body image/
26 (body adj3 (accept* or dissatisf* or image or satisf* or appreciat* or esteem)).tw.
27 eating disorder/ or anorexia nervosa/ or binge eating disorder/ or bulimia/
28 feeding behavior/
29 (bulimi* adj3 symptom*).tw.
30 (disorder* adj3 eat*).tw.
31 (emotion* adj3 eat*).tw.

32 (diet* adj4 restrain*).tw.
33 (binge adj3 eat*).tw.
34 extreme weight loss.tw.
35 loss of control.tw.
36 drive for thinness.tw.
37 ((weight or shape or eat*) adj3 concern).tw.
38 25 or 26 or 27 or 28 or 29 or 30 or 31 or 32 or 33 or 34 or 35 or 36 or 37
39 randomized controlled trial/ or controlled clinical trial/
40 (randomi?ed controlled trial* or RCT* or (controlled adj3 trial)).mp.
41 randomi?ed.ti.
42 randomly.ab.
43 trial.mp.
44 clinical trial.mp.
45 39 or 40 or 41 or 42 or 43 or 44
46 4 and 24 and 38 and 45

Database: APA PsycInfo
Search Strategy:
----
1 Obesity/
2 Overweight/
3 obes*.tw.
4 overweight.tw.
5 1 or 2 or 3 or 4
6 weight loss/ or weight control/
7 diets/
8 exp bariatric surgery/
9 exp exercise/
10 (diet* adj2 therap*).tw.
11 bariatric*.tw.
12 (low adj3 (energy or calor*) adj4 diet).tw.
13 ((pharma* or diet* or obes* or lifestyle or behavio*) adj3 (interven* or treat* or therap*)).tw.
14 ((calori* or diet*) adj3 (reduc* or restrict*)).tw.
15 (weight adj3 (manag* or los*)).tw.
16 exercis*.mp. or physical activit*.tw.
17 HAES.mp.
18 health at every size.mp.
19 (weight adj2 neutral).mp.
20 nondiet.mp.
21 (non adj2 diet).mp.
22 (intuitive adj2 eat*).mp.
23 mindful*.tw.
24 6 or 7 or 8 or 9 or 10 or 11 or 12 or 13 or 14 or 15 or 16 or 17 or 18 or 19 or 20 or 21 or 22 or 23

25 Body Image/
26 (body adj3 (accept* or dissatisf* or image or satisf* or appreciat* or esteem)).tw.7088)
27 eating disorders/ or anorexia nervosa/ or binge eating disorder/ or bulimia/ or hyperphagia/ or "purging (eating disorders)"/
28 eating behavior/ or binge eating/ or dietary restraint/
29 (bulimi* adj3 symptom*).tw.
30 (disorder* adj3 eat*).tw.
31 (emotion* adj3 eat*).tw.
32 (diet* adj3 restr*).tw.
33 (binge adj3 eat*).tw.
34 extreme weight loss.tw.
35 loss of control.tw.
36 drive for thinness.tw.
37 ((weight or shape or eat*) adj3 concern).tw.
38 25 or 26 or 27 or 28 or 29 or 30 or 31 or 32 or 33 or 34 or 35 or 36 or 37
39 randomized controlled trials/ or clinical trials/ or randomized clinical trials/
40 (randomi?ed controlled trial* or RCT* or (controlled adj3 trial)).mp.
41 randomi?ed.ti.
42 randomly.ab.
43 trial.mp.
44 clinical trial.mp.
45 39 or 40 or 41 or 42 or 43 or 44
46 5 and 24 and 38 and 45

****

EDIT intervention deconstruction framework and definitions

Supplementary File S3: EDIT intervention coding framework.

Delivery features

1. Why - Theory

**Why – Theory**		
1.1	Weight maintenance	Intervention focuses on/aims for weight maintenance but not weight loss. For example, the intervention is designed to prevent further weight gain or keep participants at designated weight or BMI z-score. Weight loss or decrease in body mass is not the target.
1.2	Weight loss	Intervention focuses on decreases in body mass. For example, implementing specific diets / intervention strategies to induce weight loss or decreases in body mass or decrease BMI z-score. Although weight maintenance may be a focus following the initial period of weight loss the target of the intervention is first to decrease body mass.
1.3	Weight loss and maintenance	Intervention has two parts, each with a focuses on decreases in body mass AND weight maintenance. For example, implementing specific diets / intervention strategies to induce weight loss or decreases in body mass or decrease BMI z-score. Weight maintenance must be a focus at some stage of the intervention following the initial period of weight loss or decrease in body mass as the target of the intervention.

2. Psychological theory or framework underpinning intervention

**Psychological theory or framework underpinning intervention**		
2.1	Cognitive behaviour therapy (CBT)	
2.2	Enhanced Cognitive Behaviour Therapy (CBT-E)	
2.3	Acceptance and Commitment therapy (ACT)	
2.4	Dialectical Behaviour Therapy (DBT)	
2.5	Family-based therapy (FBT)	<u>Do not code</u> family-based treatment of childhood obesity.
2.6	Interpersonal Therapy (IPT) for binge eating	
2.7	Trauma-informed care	
2.8	Compassion focussed therapy	
2.9	Motivational interviewing	
2.10	Other General theory (not based on psychological theory)	Extract theory name reported in intervention. For example, general behaviour change theories such as Social Cognitive Theory, Theory of Planned Behaviour, Health Belief Model, or intervention design frameworks such as intervention mapping, Behaviour Change Wheel.

3. Target population/ recipient of the intervention – AGE GROUP

**Target population/ recipient of the intervention – AGE GROUP**		
3.1	Adolescent	As reported by the trial, or when participants are aged 10-18 years
3.2	Adult	As reported by the trial, or when participants are aged 10-18 years
	Extracted except	*Extract trial reported age range.*

4. Target population/ recipient of the intervention – WEIGHT Category

**Target population/ recipient of the intervention – WEIGHT Category**		
4.1	Overweight	As reported by the trial, or when participants have a BMI of greater than or equal to 25 kg/m2 or BMI z-score of 1-2 or between the 85th-95th BMI percentile.
4.2	Obesity	As reported by the trial, or when participants have a BMI of greater than or equal to 30kg/m2 or BMI z-score greater than 2 or ≥95th BMI percentile.

4.3	Severe Obesity	As reported by the trial, or when participants have a BMI of greater than or equal to 40kg/m2 Or BMI z-score of 3 or ≥99th BMI percentile. Note in older trials may be reported as morbid obesity.<br>Note: if states obesity and doesn't provide an upper cut off, assume this also includes severe obesity.<br>*Coding plan: click maybe if unclear and check with trialist (e.g. 100% overweight based on comparisons of the body mass index (BMI = kg/m2) to the 50th percentile BMI for age and sex)*
	Extract	Code weight target category as per trial reported category and extract trials definition for each category as relevant.

5. Target population/ recipient of the intervention – *Individual vs with support person/s*

**Target population/ recipient of the intervention – Individual vs with support person/s *(code only 1 recipient)***		
5.1	Individual	A person with overweight or obesity participates in the intervention by themselves
5.2	Individual with support person	For example, parent(s) complete intervention with participant (family based treatment approach), or partners or friend attends the intervention with the participant. Do not code if adolescents require parental attendance to attend meetings due to age, without a family based approach to the intervention.
5.3	Family or household-based treatment approach	Family/household approach is taken to treatment, e.g. whole of family oriented changes to diet/activity.

6. What – materials:

**What – materials**		
6.1	Information sheets/ booklets	This includes any type of written or visual printed materials (includes images). For example, fact sheets, newsletters, recipes, meal or exercise plans etc. This can include hardcopy or softcopy resources. Assumption that information sheets/ booklets are included if personalised diet plan or similiar is provided.
6.2	Food	This includes fresh foods, portioned meals and meal-ordering services (e.g. Light and Easy, Jenny Craig), provided as part of the intervention. Do not code the recommendation to eat certain foods if participants are required to purchase food items themselves, or if catering is provided for a session. Explicit statement that food is provided is required to code.
6.3	Diet monitoring materials	This includes any type of diet monitoring materials that are provided (e.g. checklists, food diary, calorie counting).
6.4	Supplements	This includes vitamins, protein powders, and other supplements to add to meals.<br>Do not code the recommendation to purchase supplements, if participants are required to purchase items themselves.
6.5	Meal replacement products	This includes any products used to substitute a standard meal, such as shakes, bars, meal replacement powders, provided as part of the intervention.<br>Do not code the recommendation to purchase and use meal replacement products, if participants are required to purchase items themselves.
6.6	Sports equipment	This includes exercise bands, weights, treadmill etc, provided to participants as part of the intervention. Do not code the recommendation to purchase sports equipment, if participants are required to purchase items themselves.
6.7	Fitness/ activity tracker	This includes physical pedometers or accelerometers provided to participants as part of the intervention to track activity. Includes paper based tracking/diaries. Do not code the recommendation to purchase and use activity trackers, if participants are required to purchase items themselves, or if solely used as a data collection method.
6.8	Body scales	This includes 'bathroom' scales to self-monitor weight at home. Do not code the recommendation to purchase and use scales, if participants are required to purchase items themselves, or if solely used as a data collection method.
6.9	Food scales	This includes 'food' scales to measure food portions at home.<br>Do not code the recommendation to purchase and use scales, if participants are required to purchase items themselves.

6.10	Mobile app	This includes mobile apps for the intervention and used with participants as part of the intervention. Where possible note if the app was interactive/2-way communication, versus 1-way information delivery. This can include the use of activity tracker mobile applications.
6.11	Website access (online resources)	This includes websites specific for the trial and used with participants as part of the intervention. Where possible note if the website was interactive/2-way communication, versus 1-way information delivery.<br>Do not code if a website is only used for participant recruitment or to share trial materials after the study.
6.12	Social media	This includes any social media (e.g. Facebook) pages, groups or channels specific for the trial and used with participants as part of the intervention.<br>Do not code if social media is only used for participant or to share trial materials after the study.
6.13	Other materials	Please describe any other materials provided to participants as part of the intervention that are not captured in the above items.

7. What – procedures:

**What – procedures**		
Note this delivery feature category provides a high-level coding of the types of intervention content, specific details of the intervention content will be coded by the Intervention Strategies.		
7.1	Nutrition education	This includes information on food groups, daily food intakes, portion sizes, eating patterns, improving diet quality and nutrition knowledge, etc. For example, written resource provided listing servings in approximately 100 kcal portions or when dietitian reviews dietary intake, eating behaviours, and encourages adherence to the intervention.
7.2	Energy prescription or target	This includes utilising energy-based diets (e.g., Intermittent Energy Restriction) to energy intake targets to promote decrease in body mass or assist in weight maintenance.
7.3	Physical activity education	This includes information on physical activity benefits, how to schedule and perform activity.
7.4	Exercise classes	This includes practical gym classes, group activity sessions, individual personal training/ exercise physiology sessions etc, delivered as part of the intervention.
7.5	Psychological component	This includes information or education about psychological health, counselling and/or use of psychological techniques (Cognitive Behaviour Therapy, anticipatory guidance etc).

8. What – outcome measures:

**What – outcome measures**		
8.1	Weight/ adiposity	This includes measurement of weight, waist circumference, body composition etc.
8.2	Physical health outcomes	This includes measurement of a range of physical health outcomes, e.g. blood pressure, blood test, fitness.
8.3	Psychosocial /Mental health outcomes:	This includes measurement of any psychosocial or mental health outcomes, e.g. depression, quality of life, self-esteem, anxiety, body image, weight-bias internalisation. Do not code eating disorder outcome measures as trials were required to include an eating disorder outcome measure to be eligible to join the EDIT Collaboration.
8.4	Eating behaviour outcomes	This refers to eating behaviours or appetite-related outcomes (e.g. DEBQ, TFEQ). Do NOT code eating disorders or disordered eating behaviours (e.g. binge eating, EDE-Q, , CHEAT, EAT, EDI).
If measuring weight:		
8.5	Individual weighing at visits	This includes any weighing that is performed in private in an intervention, e.g. participant measured at the beginning of each one-on-one dietary counselling session. Assumption that individual weighing is conducted unless it is stated clearly otherwise.
8.6	Blind weighing at visits	This includes any time a participant is weighed with the measure recorded without sharing the weight measure with the participant. This does not refer to trials having a blinded outcome assessor.
8.7	Group weighing	This includes weights measured in a group context of more than one participant.

8.8	Communication about the ability to decline weight or opt out of weighing during visits	This includes any written or verbal communication with participants that weighing was voluntary and they could choose to opt-out at any time.

9. Who provided – intervention delivered by (personnel, training, qualifications)

**Who provided – intervention delivered by**		
9.1	Dietitian/ nutritionist	This includes research or student dietitians.
9.2	Nurse	This includes research or student nurses.
9.3	Exercise Physiologist/ Physiotherapist/ Personal trainer/ Other exercise professional	This includes research or student exercise professionals.
9.4	Psychologist/ counsellor	This includes research or student psychologists.
9.5	Physician – pediatrician, GP, endocrinologist	This includes any medical specialists and research or student physicians.
9.6	Pharmacist	This includes research pharmacists.
9.7	Researcher/ non-health professional	This includes researchers or individuals who do not have a health professional background.
9.8	Self-delivered	No researcher/health professional contact during the intervention (e.g. self-help program, application, website-based intervention).<br>Excluding a once-off session/contact to explain the self-delivered content.
9.9	Other, free text	Please describe other professionals who are involved in the intervention delivery not listed above (e.g. school teacher).

10. Training for interventionist

**Training for interventionist**		
10.1	Training for the interventionist	Was specific training provided to intervention facilitators
10.2	Describe	If yes, briefly describe the type and duration of the training.

11. How – delivery mode

**How – delivery mode**		
11.1	Face-to-face	In person delivery.
11.2	Computer/ Web-based / Online	Website, mobile application, emails etc.
11.3	Call (telephone, video)/ SMS	Any form of phone or video calls including appointments, follow up phone calls/texts, text-based intervention delivery.
11.4	Printed material	Handouts, workbooks, meal plans, recipes etc.
*In additional to the above coding, provide the summary level coding of the intervention delivery. **<u>Code all that apply</u>**, for example there may be initial individual session, followed by a series of group sessions.*		
11.5	Individual	Delivery on individual / one-on-one basis, e.g., individual person, family (parent/child). Assumption that the delivery mode is individual if not stated otherwise.
11.6	Group	Delivery is to a group (any sessions with more than one participant attending).<br>Note: this can include any aspect that is delivered to a group of participants including online forums.
11.7	Peer support	Intervention delivery to the participant who has a peer support worker as part of the intervention (.e. intervention links the participant with a peer support worker)<br>*Do not code* if participant is encouraged to bring a support person to the intervention (i.e. code 5.2)

12. Where – intervention setting:

**Where – intervention setting**		
*Refers to where the clinician and participant meet or interact. Do not code intervention setting if only used for recruitment, e.g. participants recruited through hospital inpatient setting but then all intervention sessions were delivered through outpatient setting. Author location or address cannot be used to determine intervention setting. **<u>Code all setting relevant for the participant intervention setting.</u>***		
12.1	Hospital outpatient	Attending a hospital facility as an outpatient for the intervention (clinic, university hospital etc).
12.2	Hospital inpatient	Participants receiving intervention components while an inpatient in a hospital setting.

12.3	University / research centre	Any university or research institute setting.
12.4	Primary care	Any primary health care setting such as GP clinic, medical centre etc.
12.5	Community	Any community-based settings, such as community centres, support groups, local government etc.
12.6	School	Any formal education setting such as primary school or high school, including after hours care settings.
12.7	Household residence	Any component of the intervention that is delivered in a participants home, such as home visits.
12.8	Virtual	Receiving intervention through computer/mobile app/online or call/SMS components.
12.9	Commercial provider centres	Any commercial provider offices such as Weight Watches, Jenny Craig, Slimming World etc where participants attend the setting.
12.10	Workplace	Any workplace that the intervention is delivered via, e.g. corporate programs, military.

13. When and how much (intervention dose)

**When and how much (intervention dose)**		
13.1	Total number of contacts	Record total number of intervention sessions during intervention (not follow-up period). Record number and duration of contacts in extracted excerpts.
13.2	Overall intervention duration (weeks)	Total length of the intervention period (in weeks). The intervention period is defined as the time when any contact/ advice is provided by study personnel and includes weight loss and weight maintenance periods but excludes follow-up period where there is no ongoing contact with the study team (excluding solely data collection). 'Keeping in touch' strategies such as newsletters to encourage retention at follow-up, but that do not provide advice, will not be considered part of the intervention.
13.3	Total duration of contacts/sessions (minutes)	Sum of duration of all intervention sessions. Select unclear if no information provided or if intervention does not have any verbal form of contact with participants (e.g. text-messaging, online video education only interventions). Document anything that is reported re: time.<br><br>*Note: when unclear is selected – check with Trialist to see if they can provide an estimate*
13.4	Intensity	This relates to the frequency of the intervention sessions, code as less than weekly, weekly, every 2-3 weeks, monthly, >monthly, staged approach (weekly to >monthly).<br><br>*Note: select only one, I.e. if different frequencies select staged approach.*

14. Post-intervention support

**Post-intervention support**		
14.1	Referral to other services	At the end of the end of the intervention (i.e., end of contact with study personnel for intervention, not data collection) , participants are referred to other appropriate services (e.g. referred to local dietitians for ongoing support).
14.2	Additional information provided	At the end of the end of the intervention (i.e. end of contact with study personnel for intervention, not data collection period), participants are provided with addition information (verbal or written) (e.g. advice on healthy eating)
14.3	Other	At the end of the intervention (i.e. end of data collection period), is there any other support providing (other than referrals to other services or additional information).

15. Tailoring

**Tailoring**		
15.1	Was there an element of tailoring in the intervention?	Tailoring refers to if the intervention was planned to be personalised, titrated or adapted at the participant level. For example, a personalised meal plan to suit the individuals lifestyle and dietary preferences. If tailoring at the participant level was planned code as yes and briefly describe / extract text explaining how the intervention will be/was tailored. Do not code if energy

		levels are based on age/sex, or if the intervention only includes individual delivery or goal setting.<br><br>*Note: only code if the trial explicitly states personalisation or tailoring.*
15.2	Who or which subgroup was the intervention tailored to?	Record if the tailoring was for 'all' participants, or to certain subgroup (e.g., only those rated as having low for self-esteem received a personalised self-efficacy package)

16. Modifications

**Modifications**		
16.1	Was the intervention modified?	Modifications refers to if the intervention was modified during the study at the intervention level, i.e. changed from what was planned in the intervention protocol. This may have been due to changes required with COVID-19 restrictions or a range of other reasons. If the intervention was modified at the intervention level code as yes and briefly describe / extract text explaining how the intervention was modified. Modifications will not be reported in published trial protocols.<br><br>The 'Intervention Coding Request Form' will ask Trial Representative to reported modifications to the intervention; this will be used to cross-check modifications coded from the materials received.

17. Fidelity

**Fidelity**		
17.1	Were any intervention fidelity procedures (actual or planned) described?	Planned fidelity relates to proactive steps to ensure and/or monitor fidelity of the intervention; this may be described in the trial protocol as it relates to the planned processes/methods that will be used. For example, standardised training and facilitator manuals, weekly facilitator meetings, independent observations of intervention delivery, checklist of key messages delivered. The key to planned fidelity is the intentional purpose to ensure the intervention is delivered consistently as it was developed to be delivered.<br><br>Actual fidelity relates to measures / outcomes of fidelity reported based on the actual delivery of the intervention, for example the number of sessions attended by all participants. Actual fidelity will not be reported in published trial protocols. For example, rating by an independent experienced observer using a standardised process found that all intervention messages were consistently delivered to participants. This may not be reported in detail, a results paper may report that the planned activities to ensure fidelity were completed but not report the findings or outcomes of that.

Delivery strategies

Intervention intent, framing and outcomes

18. Framing of the intervention (communication strategies)

**Framing of the intervention (communication strategies)**		
<u>*Note this information will be trialist reported*</u>		
18.1	Education provided on obesity as a disease	Information was provided to participants on risks associated with obesity, in particular the implications of obesity on health and quality of life. Describing obesity as a disease indicates that it is not a personal choice and may include discussion on physiology, metabolism etc
18.2	Education that weight loss is required to improve health outcomes	Session one highlights individuals weight loss target needed to improve insulin sensitivity and blood lipid levels. Discusses the association between weight and several health conditions.
18.3	Education that health outcomes are not dependent on weight	Group sessions discussed the association between behaviours and health outcomes, highlighting that exercise can improve cardiometabolic health without needing weight loss.

18.4	Education that health behaviours are linked to health outcomes	Participants are given leaflets that highlight improvements to diet quality and exercise can reduce cholesterol and blood pressure. The mobile application included topics that discussed the effectiveness of diet and exercise on improving mobility and fitness.
18.5	Feedback on change in metabolic health outcomes (e.g., insulin sensitivity, cholesterol levels)	Participants cholesterol levels were reported to them by the GP, at week 5 of the 10 week intervention. Do not code Data collection as this does not constitute feedback. Feedback must be on a change and be part of the intervention to change behaviour.
18.6	Language	***Note: to be coded by trialists:***<br>○ Predominately weight focused language<br>○ Predominately health focused language<br>○ Combination weight and health language

19. Outcome related strategies

**Outcome related strategies**		
19.1	Encourages weight focused goals	This refers to specific weight-loss targets that are communicated to participants (cannot assume this based on overarching weight loss intention of trial alone). E.g. Participants set specific, weight orientated goals with the physician at their first visit (e.g. 1kg/week). These were observed for the duration of the intervention.
19.2	Discourages weight-focused goals (instead focused on health-related goals)	At the beginning of the group session, each participant set a goal to improve aspects of their health using the SMART goal setting technique.
19.3	Feedback on weight change during the intervention	Participant's weight was measured at the beginning of each GP visit, and plotted on a chart in participant's workbook. Results were discussed with the participant.
19.4	Feedback on other measures of weight adiposity (e.g. body composition, waist circumference)	Participant's waist circumference was measured at the beginning of each GP visit, and results were discussed with the participant.
19.5	Promotes weight loss rewards or incentives	Once participants had reached their goal weight, they were given a $50 gift card
19.6	Encourages self-monitoring of weight (e.g. self-weighing at home)	Participants were given scales and a weight loss chart to perform self-weighing at home.
19.7	Discourages home weighing or frequent weighing	Group facilitators told participants to avoid weighing at home (i.e. limit to once a week at the group weight measures), throughout the program.

Dietary strategies

20. Nutrition education

**Nutrition education**		
20.1	Education on portion size (e.g. portion plate model, serve sizes, etc)	Topics for the first month included correct portion/serve sizes, food groups, and daily dietary intakes for adults.<br>Note: Also code yes for 'Education on healthy eating guide'
20.2	Education on label reading	The facilitator brought in different packaged foods and taught participants how to read nutrition labels on each item.
20.3	Education on metabolism	At month 3, group discussion involved metabolisms, how the body stores/uses energy from the foods we eat, and individual differences in energy use and storage.
20.4	Education on healthy eating guide (e.g. promotes balanced meals and food groups)	The session focused on the healthy eating guide and discussed recommendations as to the average number of standard serves of the five core food groups an individual should consume in to meet their nutritional requirements.
20.5	Education on energy/ macronutrient (e.g. fat, sugar) content of foods	The video for week 5 was recorded by a registered Nutritionist, who discussed the different macronutrients in foods.
20.6	Categorisation of foods as good versus bad (e.g. traffic light system; defines foods as good vs bad (e.g treat/ sometimes foods)	Handout 1 includes a guide of which foods to increase and which foods to limit. This refers to framing of food as good versus bad

20.7	Provides cultural adaptations relating to diet	Participants were given reprieve from their meal plans during Cultural Celebrations (e.g., Ramadan).<br>*Note: 'Adapting to individual preferences or education healthy eating guide' is not sufficient to code, needs to refer to inclusion or exclusion of foods in meal plan based on cultural preferences etc to be coded.*

21. Dietary self-monitoring

**Dietary self-monitoring**		
21.1	Dietary self-monitoring – food based (e.g. food diary, points system)	Each food on the meal plan came with corresponding points. Participants recorded the points consumed during the day in an online application.
21.2	Dietary self-monitoring – energy based (e.g. calorie counting)	Participants receive a detailed meal plan with a variety of food choices and are encouraged to monitor intake using a calorie counting smartphone application.
21.3	Dietary self-monitoring – weighing food	Participants were recommended to weigh their food to help with portion control.
21.4	Review/feedback on self-monitoring (e.g., feedback on food diary)	The study app allowed participants to record their food diary and receive feedback on their total energy intake from the food consumed each day. External review e.g. diet history is not applicable.<br>*Note: would also code as 'Dietary monitoring – food based'*

22. Dietary prescription

**Dietary prescription**		
22.1	Hypocaloric diet (reduced calorie diet)	Participants were prescribed a hypocaloric diet that would reduce their current energy intake (e.g. reduce by 500kcal) or a set calorie amount e.g. 1500kcal/day.<br>Notes: Record details of the set calorie amount or reduction. Can assume yes if only individual dietary prescription is stated and no other specific strategy is mentioned – ask trialists to check
22.2	Traffic light diet (categorising foods as red, yellow, green)	Participants were instructed to follow a traffic light diet throughout the intervention period.
22.3	Intermittent energy restriction/ intermittent fasting (chrononutrition)	All participants in the intervention group were prescribed an intermitted energy restriction of <600 kcal for 2 days per week.
22.4	Macronutrient prescription (e.g. low-carbohydrate, high-protein)	Participants in the intervention group follow a high protein, low carbohydrate diet.
22.5	Ketogenic diet	Participants were instructed to follow a ketogenic diet.
22.6	Very low energy diet (VLED/ VLCD - restrictive calorie restriction e.g. 800-1000 kcal/ day)	Participants were prescribed a VLED that would reduce their current energy intake to 800kcal per day.

23. Delivery of the dietary intervention

**Delivery of the dietary intervention**		
23.1	Prescriptive/ specific meal plan (external control)	Participants were provided a specific meal plan, detailing the types, amounts and eating occasions to consume each day (e.g. only fruit at morning tea).
23.2	Flexible meal plan (provides choice, ownership over dietary intake)	Participants receive a flexible meal plan builder containing detailed information on a wide variety of food choices that they can construct a weekly meals list.
23.3	Use of meal replacement products – partial or full	Participants are to eat 3 meals a day, with 2 of these replaced with either a meal replacement bar or shake.
23.4	Promotes 'free' foods, ad-lib intake of certain foods	On energy restricted days participants can also eat ad libitum from a list of "low-energy" fruits and vegetables in addition to the energy prescription.

24. Dietary behaviour change strategies

**Dietary behaviour change strategies**		
24.1	Problem solving barriers to dietary change	Participants wrote down a list of potential barriers to meeting their dietary goals, and ways to manage or avoid these were discussed as a group. Participants then picked three solutions they would use when facing these problems.

24.2	Feedback on dietary behaviours (e.g. diet history at visits)	At visit 1, the dietitian will conduct a diet history and provide feedback on areas to improve adherence with dietary guidelines.
24.3	Encourages dietary focused goals with/ without review	Participants set goals to change their diet, focus was given to small and achievable goals, which were reviewed each visit.
24.4	Shopping support (planning, product choice, family/ partner involvement in food purchases)	Participants and their support person are encouraged to work together to develop a shopping list guided by the dietitian.
24.5	Addresses home/food environment (e.g. identifying triggers, permissive vs restrictive environment; stimulus control)	Participants were given a pantry layout guide to restructure foods in easy reach (i.e. lowest energy choices) and identify food to remove from the house.
24.6	Addresses food/meal preparation skills (e.g. cooking demonstrations, recipes)	Participants came to cooking sessions twice a month, where they learnt how to create healthy, nutritious meals at home.

Eating behaviours/ disordered eating

25. Addresses disordered eating

**Addresses disordered eating**		
25.1	Identifies disordered eating behaviours (e.g. binge eating, emotional eating, secret eating, guilt related to eating, loss of control over eating)	Before commencing, participants were screened for disordered eating behaviours. These will be addressed throughout the program.<br>Note: cannot assume this occurs as part of the intervention if it is an outcome measure
25.2	Explores individual underlying causes/ drivers of disordered eating (e.g. teasing/ bullying, trauma, body image disturbance/pre-occupation with weight and shape, emotional regulation)	Psychologists visited adolescents once a month at the residence to discuss weight-related bullying experienced at school.
25.3	Addresses disordered eating behaviours and cognitions (e.g. identifying triggers, strategies to prevent emotional eating, over-focus on energy expenditure)	Participants reflected on triggers for their disordered eating behaviours and discussed ways to minimise the frequency of triggers. E.g. binge eating was a topic during group discussions
25.4	Education on risk of eating disorders	Education is provided on the risk of eating disorders. E.g. Parents are educated on red flags/ disordered eating behaviours (e.g. hiding food) or participants were educated on harms of excessive restriction (e.g. on menstrual cycle).<br>Note: study information sheets mentioning risks of eating disorders are not considered part of the intervention.

26. Promotes healthful/ helpful eating behaviours

**Promotes healthful/ helpful eating behaviours**		
26.1	Promotes mealtime routines (e.g. regular meals, avoid meal skipping)	Participants were given a handout identifying the importance of eating regular meal and created a personalised meal routine or meal plan.<br>*Note: Code this item if 'Prescriptive/ specific meal plan (external control)' or 'Flexible meal plan (provides choice, ownership over dietary intake)' are coded.*
26.2	Promotes meal time support (e.g. support while eating, family meals, social eating)	Parents were encouraged to have family meals whenever possible, where they can consume evening meals together with adolescents.

26.3	Addresses meal time environment	Participants were encouraged to create a positive mealtime environment including no access to phones or tv, and eating at the table.
26.4	Increasing awareness of hunger/ fullness/ satiety	Participants were encouraged to keep a diary noting their hunger and satiety for the day.
26.5	Encourages mindful eating principles or practice (e.g. avoiding distractions while eating)	Mobile phones and televisions were encouraged to be turned off or put away during meal times. (Note to code it must have label of “mindful eating”.)
26.6	Encourages intuitive eating principles or practice (e.g. Promotes anti-diet, hunger and fullness, food enjoyment, body respect)	Session two included provided tips to practice intuitive eating. (Note to code it must have label of “intuitive eating”.)

Movement and sleep related strategies

27. Physical activity education

**Physical activity education**		
27.1	Education to increase physical activity (e.g. staged introduction of activity, suggested activities)	The intervention included information on different activities to try to increase physical activity.<br>Note: if providing feedback or advice on physical activity there is an assumption this will include education, therefore code this item if coding ‘External feedback on self-monitoring'.
27.2	Promotes joyful movement and activity	Participants were given access to a variety of exercise classes to select the most enjoyable for them and improves quality of life. Note mindfulness applied to exercise can include focus on joyful movement
27.3	Encourages strict/ formal activity plan (e.g. gym program)	Participants were given a detailed gym program that gradually increased activity.
27.4	Education on Non Exercise Activity Thermogenesis (NEAT) (energy expended during tasks of daily living)	In week 8, group discussion centred on how housework and office work can also expend energy, and how it can help/limit weight loss.
27.5	Provides cultural adaptations relating to physical activity	Physical activity programs were individualised to provide culturally safe activity suggestions.

28. Physical activity prescription

**Physical activity prescription**		
28.1	Provides a prescriptive exercise plan	Each participant was given an individualised exercise plan that structured time for 30 mins of exercise and 10,000 steps daily for 6 weeks.
28.2	Provides flexible exercise plan (e.g. suggested activities, encouraging choice)	Participants were given a flexible exercise plan including various potential exercise plans and activities that participants could choose from each day.
28.3	Provides supervised group exercise classes/ program	At the end of each education session, participants attended fitness classes together, instructed by students completed their cert IV in fitness.
28.4	Provides individual personal training	Participants attended one-on-one Personal Trainer sessions once a week for the first 6 months.

29. Physical activity monitoring

**Physical activity monitoring**		
29.1	Self-monitoring of activity (e.g. diary, pedometer)	Participants were instructed to wear a pedometer every day to monitor activity levels, and record steps in their activity diary.
29.2	External feedback on self-monitoring (e.g., feedback on exercise diary, step count)	Feedback on step count is given by the exercise physiologist each visit.

30. Behaviour change strategies related to physical activity

**Behaviour change strategies related to physical activity**		
30.1	Encourage activity focused goals (including time, duration, mode) with/ without review	Participants set physical activity goals using SMART goal setting.

30.2	Increasing skills to undertake physical activity (e.g. demonstration of activity such as pictures/ videos/ live demos, help with scheduling/ planning for activity)	Instructional videos on how to perform exercises at home were accessible on the study website.
30.3	Feedback on physical activity behaviours and/ or change in fitness	Personal Trainers provide feedback on technique and improvements at the end of each session.
30.4	Problem solving barriers to physical activity	Potential barriers to engaging physical activity were addressed, with participants creating action plans. May include exploring motivation for physical activity.

31. Addressing sedentary time

**Addressing sedentary time**		
31.1	Education to reduce/ limit sedentary time (e.g. screen time)	Pamphlets outlining the consequences of sedentary time were provided.
31.2	Sedentary time focused goals (time, duration, mode) with/ without review	Participants set goals to reduce daily sedentary time and were given stickers by their GP at the end of the week for successful days.
31.3	Encourages self-monitoring of sedentary time (e.g. screen time monitoring, setting app limits, reminders to stand)	Participants set movement reminders on their smart watches if movement had not been detected in an hour.
31.4	Problem solving barriers to reducing sedentary time	Participants reflected on their daily routines and created a list of ways to reduce sedentary times and activities to promote movement.

32. Addressing sleep health

**Addressing sleep health**		
32.1	Education on sleep health (e.g. duration, quality, routine)	Leaflets outlining the recommended hours of sleep for adolescents as well how to create a sleep schedule were given in the handout.<br>A "sleep health" checklist was given to participants to ensure a good sleep environment was created (e.g. limit screen time before bed)
32.2	Sleep health focused goals with/without review	Participants set goals to aim for 8 hours of sleep daily.
32.3	Encourages self-monitoring of sleep (e.g. sleep diary)	Participants wore smart watches which recorded sleep times and stages.
32.4	Problem solving barriers to improving sleep health	Participants reviewed their sleep routine with clinicians and discussed strategies to overcome barriers.<br>Note: This may include feedback and strategies to address sleep health e.g. participants were referred to medical practitioners for treatment of obstructive sleep apnoea

Psychosocial health related strategies

33. Addresses mental health conditions e.g. depression, anxiety, PTSD

**Addresses mental health conditions e.g. depression, anxiety, PTSD**		
33.1	Identifies mental health condition	The first session with the psychologist focussed on identifying any mental health conditions.
33.2	Provides referral for psychological support	The intervention was delivered by a dietitian and exercise specialist, where required referrals were provided for psychological support. (Note code any type of referral e.g. internal or external of the intervention)
33.3	Addresses mental health condition within the intervention	Optional sessions were provided between session 1 and 2 to address underlying mental health conditions identified in session 1.
33.4	Addresses self-esteem	The intervention includes activities to improve self-esteem.

34. Addresses body image

**Addresses body image**		
34.1	Addresses body image concerns	Individual sessions allow the intervention facilitator to discuss body image concerns in a supportive space.

34.2	Education on the role of social media (e.g. media literacy training)	In the group session, participants learn about the impact of social media on body image.
34.3	Promotes body compassion/ acceptance/ positivity	The app provides weekly reminders that encourage body positivity.

35. Addresses weight stigma

**Addresses weight stigma**		
35.1	Education and/ or strategies to increase resilience to weight stigma, bullying, teasing	Session five will focus on strategies to increase resilience to weight related stigma and teasing.<br>Note: This relates to the individual participating in the intervention.
35.2	Addresses weight-focused communication skills (e.g. how to communicate with peers/ family about weight, how to address weight-related comments from peers/ family)	The intervention includes tips for how address weight-related comments from peers and family members.
35.3	Education to support network persons on weight stigma/teasing	Information sheets on weight stigma and teasing were provided to parents and participant's schools.

36. Psychosocial health related monitoring

**Psychosocial health related monitoring**		
36.1	Self-monitoring of thoughts, feelings, mood (e.g. mood diary)	Participants were told to write in their mood journal any time an unhelpful thought came to mind.
36.2	Review/feedback on self-monitoring (e.g. mood diary)	Mood journals were shared with the intervention facilitator to review and provide feedback on changes in their mood.
36.3	Encourages self-assessment of overall wellbeing (e.g. reflective practice)	Once a week, participants completed a brief questionnaire to assess their overall wellbeing by reflecting on experiences of the past week.

37. Behaviour change strategies related to psychosocial issues

**Behaviour change strategies related to psychosocial issues**		
37.1	Encourages psychosocial health related goals with/ without review	Participants set goals to maintain mental health and strong relations with others.
37.2	Increases skills to manage psychosocial health (e.g. stress management)	Participants worked with counsellors to outline stressors and triggers that lead to emotional eating and provide tools to manage it. Note: Would also code the strategy 'Addresses disordered eating behaviours'
37.3	Inclusion of peer/ social support strategies	Participants are encouraged to share their goals with family or partner. Group sessions were designed to provide social support between participants in the intervention.

Supplementary File S4: Sensitivity Analysis including all Eligible Trials using Published Intervention Descriptions.

Analyses consisted of 29 studies with 65 intervention arms (k). This included original coding of all studies in primary analyses as well as 6 additional studies that could not be verified.[1-9]

Delivery Features

Cluster	Components	Frequency in intervention arms (%) k=65
**Why - Theory**	Weight maintenance	5 (8)
	Weight loss	40 (62)
	Weight loss and maintenance	19 (29)
**Psychological theory or framework underpinning intervention**	Cognitive behavioural therapy (CBT)	23 (35)
	Enhanced Cognitive Behavioural Therapy (CBT-E)	0 (0)
	Acceptance and Commitment therapy (ACT)	1 (2)
	Dialectical Behaviour Therapy (DBT)	0 (0)
	Family-based therapy (FBT)	8 (12)
	Interpersonal Therapy (IPT) for binge eating	3 (5)
	Trauma-informed care	0 (0)
	Compassion focussed therapy	0 (0)
	Motivational interviewing	10 (15)
	Other General theory (not based on psychological theory)	18 (28)
**Target population/recipient of the intervention – AGE GROUP**	Adolescent	65 (100)
	Adult	4 (6)
**Target population/recipient of the intervention – WEIGHT Category**	Overweight	54 (83)
	Obesity	62 (95)
	Severe Obesity	50 (77)
**Target population/recipient of the intervention – Individual vs with support person/s**	Individual	14 (22)
	Individual with support person	48 (74)
	Family or household-based treatment approach	3 (5)
**What – materials**	Information sheets/booklets	40 (62)
	Food	8 (12)
	Diet monitoring materials	21 (32)
	Supplements	2 (3)

	Meal replacement products	2 (3)
	Sports equipment	0 (0)
	Fitness/activity tracker	9 (14)
	Body scales	8 (12)
	Food scales	3 (5)
	Mobile app	5 (8)
	Website access (online resources)	6 (9)
	Social media	0 (0)
	Other materials	5 (8)
**What – procedures**	Nutrition education	60 (92)
	Energy prescription or target	26 (40)
	Physical activity education	56 (86)
	Exercise classes	14 (22)
	Psychological component	41 (63)
**What – outcome measures**	Weight/adiposity	65 (100)
	Physical health outcomes	19 (29)
	Psychosocial/Mental health outcomes:	46 (71)
	Eating behaviour outcomes	11 (17)
	Individual weighing at visits	65 (100)
	Blind weighing at visits	0 (0)
	Group weighing	0 (0)
	Communication about the ability to decline weight or opt out of weighing during visits	0 (0)
**Who provided – intervention delivered by (personnel, training, qualifications)**	Dietitian/nutritionist	28 (43)
	Nurse	2 (3)
	Exercise Physiologist/Physiotherapist/Personal trainer/Other exercise professional	8 (12)
	Psychologist/counsellor	36 (55)
	Physician – paediatrician, GP, endocrinologist	13 (20)
	Pharmacist	0 (0)
	Researcher/non-health professional	9 (14)
	Self-delivered	3 (5)
	Other	9 (14)
**Training for interventionist**	Training for the interventionist	31 (48)
**How – delivery mode**	Face-to-face	57 (88)
	Computer/Web-based/Online	7 (11)
	Call (telephone, video)/SMS	15 (23)
	Printed material	22 (34)
	Individual	52 (80)

	Group	37 (57)
	Peer support	5 (8)
**Where - Intervention setting**	Hospital outpatient	16 (25)
	Hospital inpatient	5 (8)
	University/research centre	13 (20)
	Primary care	8 (12)
	Community	6 (9)
	School	1 (2)
	Household residence	0 (0)
	Virtual	8 (12)
	Commercial provider centres	1 (2)
	Workplace	0 (0)
**Post-intervention support**	Referral to other services	2 (3)
	Additional information provided	2 (3)
	Other	2 (3)
**Tailoring**	Was there an element of tailoring in the intervention?	31 (48)
**Modifications**	Was the intervention modified?	3 (5)
**Fidelity**	Were any intervention fidelity procedures (actual or planned) described?	33 (51)

Delivery Features – Intervention Dose

**When and how much (intervention dose)**	**Median**	**Range**
Total number of contacts	16.5[a]	1 - 146
Overall intervention duration (weeks)	25[b]	0.14 - 86
Total duration of contacts/sessions (minutes)	1080[c]	36 - 3600 (0.6 - 60 hours)

[a]13/65 (20%) could not be calculated
[b]1/65 (1.54%) could not be calculated
[c]32/65 (49.23%) could not be calculated

Delivery Features – Intervention Intensity

**Intensity**	**Frequency in intervention arms (%) k=65**
Less than weekly	9 (14)
Weekly	18 (28)
Every 2-3 weeks	3 (5)
Monthly	1 (2)
Greater than monthly	0 (0)
Stage approach (e.g. weekly>monthly)	32 (49)
Unable to calculate	2 (3)

Intervention Strategies

Cluster	Components	Frequency in intervention arms (%) k=65
**Intervention Intent, Framing and Outcomes**		
Framing of the intervention (communication strategies)	Education provided on obesity as a disease	5 (8)
	Education that weight loss is required to improve health outcomes	3 (5)
	Education that health outcomes are not dependent on weight	2 (3)
	Education that health behaviours are linked to health outcomes	3 (5)
	Feedback on change in metabolic health outcomes (e.g., insulin sensitivity, cholesterol levels)	3 (5)
	**Interventions with any strategy in this cluster**	**10 (15)**
Outcome related strategies	Encourages weight focused goals	35 (54)
	Discourages weight-focused goals (instead focused on health-related goals)	7 (11)
	Feedback on weight change during the intervention	22 (34)
	Feedback on other measures of weight adiposity (e.g. body composition, waist circumference)	2 (3)
	Promotes weight loss rewards or incentives	8 (12)
	Encourages self-monitoring of weight (e.g. self-weighing at home)	12 (18)
	Discourages home weighing or frequent weighing	1 (2)
	**Interventions with any strategy in this cluster**	**43 (66)**
**Dietary strategies**		
Nutrition education	Education on portion size (e.g. portion plate model, serve sizes, etc)	22 (34)
	Education on label reading	10 (15)
	Education on metabolism	0 (0)
	Education on healthy eating guide (e.g. promotes balanced meals and food groups)	44 (68)
	Education on energy/ macronutrient (e.g. fat, sugar) content of foods	22 (34)

	Categorisation of foods as good versus bad (e.g. traffic light system; defines foods as good vs bad (e.g treat/ sometimes foods)	25 (38)
	Provides cultural adaptations relating to diet	4 (6)
	**Interventions with any strategy in this cluster**	**60 (92)**
Dietary self-monitoring	Dietary self-monitoring – food based (e.g. food diary, points system)	27 (42)
	Dietary self-monitoring – energy based (e.g. calorie counting)	7 (11)
	Dietary self-monitoring – weighing food	4 (6)
	Review/feedback on self-monitoring (e.g., feedback on food diary)	25 (38)
	**Interventions with any strategy in this cluster**	**34 (52)**
Dietary prescription	Hypocaloric diet (reduced calorie diet)	28 (43)
	Traffic light diet (categorising foods as red, yellow, green)	17 (26)
	Intermittent energy restriction/ intermittent fasting (chrononutrition)	3 (5)
	Macronutrient prescription (e.g. low-carbohydrate, high-protein)	9 (14)
	Ketogenic diet	0 (0)
	Very low energy diet (VLED/ VLCD - restrictive calorie restriction e.g. 800-1000 kcal/ day)	2 (3)
	**Interventions with any strategy in this cluster**	**39 (60)**
Delivery of dietary intervention	Prescriptive/ specific meal plan (external control)	14 (22)
	Flexible meal plan (provides choice, ownership over dietary intake)	31 (48)
	Use of meal replacement products – partial or full	2 (3)
	Promotes ‘free’ foods, ad-lib intake of certain foods	4 (6)
	**Interventions with any strategy in this cluster**	**37 (57)**
Dietary behaviour change strategies	Problem solving barriers to dietary change	36 (55)
	Feedback on dietary behaviours (e.g. diet history at visits)	22 (34)
	Encourages dietary focused goals with/ without review	30 (46)
	Shopping support (planning, product choice, family/ partner involvement in food purchases)	17 (26)
	Addresses home/food environment (e.g. identifying triggers, permissive vs restrictive environment; stimulus control)	35 (54)

	Addresses food/meal preparation skills (e.g. cooking demonstrations, recipes)	19 (29)
	**Interventions with any strategy in this cluster**	**52 (80)**
**Eating behaviours/disordered eating**		
Addresses disordered eating	Identifies disordered eating behaviours (e.g. binge eating, emotional eating, secret eating, guilt related to eating, loss of control over eating)	16 (25)
	Explores individual underlying causes/ drivers of disordered eating (e.g. teasing/ bullying, trauma, body image disturbance/pre-occupation with weight and shape, emotional regulation)	7 (11)
	Addresses disordered eating behaviours and cognitions (e.g. identifying triggers, strategies to prevent emotional eating, over-focus on energy expenditure)	12 (18)
	Education on risk of eating disorders	6 (9)
	**Interventions with any strategy in this cluster**	**23 (35)**
Promotes healthful/helpful eating behaviours	Promotes mealtime routines (e.g. regular meals, avoid meal skipping)	20 (31)
	Promotes meal time support (e.g. support while eating, family meals, social eating)	16 (25)
	Addresses meal time environment	12 (18)
	Increasing awareness of hunger/ fullness/ satiety	10 (15)
	Encourages mindful eating principles or practice (e.g. avoiding distractions while eating)	4 (6)
	Encourages intuitive eating principles or practice (e.g. Promotes anti-diet, hunger and fullness, food enjoyment, body respect)	1 (2)
	**Interventions with any strategy in this cluster**	**35 (54)**
**Movement and sleep related strategies**		
Physical activity education	Education to increase physical activity (e.g. staged introduction of activity, suggested activities)	56 (86)
	Promotes joyful movement and activity	6 (9)
	Encourages strict/ formal activity plan (e.g. gym program)	11 (17)
	Education on Non Exercise Activity Thermogenesis (NEAT) (energy expended during tasks of daily living)	6 (9)
	Provides cultural adaptations relating to physical activity	0 (0)

	**Interventions with any strategy in this cluster**	**57 (88)**
Physical activity prescription	Provides a prescriptive exercise plan	17 (26)
	Provides flexible exercise plan (e.g. suggested activities, encouraging choice)	28 (43)
	Provides supervised group exercise classes/ program	16 (25)
	Provides individual personal training	6 (9)
	**Interventions with any strategy in this cluster**	**37 (57)**
Physical activity monitoring	Self-monitoring of activity (e.g. diary, pedometer)	29 (45)
	External feedback on self-monitoring (e.g., feedback on exercise diary, step count)	21 (32)
	**Interventions with any strategy in this cluster**	**29 (45)**
Behaviour change strategies related to physical activity	Encourage activity focused goals (including time, duration, mode) with/ without review	39 (60)
	Increasing skills to undertake physical activity (e.g. demonstration of activity such as pictures/ videos/ live demos, help with scheduling/ planning for activity)	17 (26)
	Feedback on physical activity behaviours and/ or change in fitness	20 (31)
	Problem solving barriers to physical activity	28 (43)
	**Interventions with any strategy in this cluster**	**44 (68)**
Addressing sedentary time	Education to reduce/ limit sedentary time (e.g. screen time)	24 (37)
	Sedentary time focused goals (time, duration, mode) with/ without review	13 (20)
	Encourages self-monitoring of sedentary time (e.g. screen time monitoring, setting app limits, reminders to stand)	11 (17)
	Problem solving barriers to reducing sedentary time	11 (17)
	**Interventions with any strategy in this cluster**	**24 (37)**
Addressing sleep health	Education on sleep health (e.g. duration, quality, routine)	11 (17)
	Sleep health focused goals with/without review	2 (3)
	Encourages self-monitoring of sleep (e.g. sleep diary)	2 (3)
	Problem solving barriers to improving sleep health	0 (0)
	**Interventions with any strategy in this cluster**	**11 (17)**
**Psychosocial health related strategies**		

Addresses mental health conditions	Identifies mental health condition	6 (9)
	Provides referral for psychological support	4 (6)
	Addresses mental health condition within the intervention	2 (3)
	Addresses self-esteem	9 (14)
	**Interventions with any strategy in this cluster**	**11 (17)**
Addresses body image	Addresses body image concerns	16 (25)
	Education on the role of social media (e.g. media literacy training)	3 (5)
	Promotes body compassion/ acceptance/ positivity	5 (8)
	**Interventions with any strategy in this cluster**	**16 (25)**
Addresses weight stigma	Education and/ or strategies to increase resilience to weight stigma, bullying, teasing	13 (20)
	Addresses weight-focused communication skills (e.g. how to communicate with peers/ family about weight, how to address weight-related comments from peers/ family)	7 (11)
	Education to support network persons on weight stigma/teasing	10 (15)
	**Interventions with any strategy in this cluster**	**13 (20)**
Psychosocial health related monitoring	Self-monitoring of thoughts, feelings, mood (e.g. mood diary)	3 (5)
	Review/feedback on self-monitoring (e.g. mood diary)	0 (0)
	Encourages self-assessment of overall wellbeing (e.g. reflective practice)	0 (0)
	**Interventions with any strategy in this cluster**	**3 (5)**
Behaviour change strategies related to psychosocial issues	Encourages psychosocial health related goals with/ without review	2 (3)
	Increases skills to manage psychosocial health (e.g. stress management)	12 (18)
	Inclusion of peer/ social support strategies	15 (23)
	**Interventions with any strategy in this cluster**	**24 (37)**

References.

1. Brennan L, Walkley J, Fraser SF, Greenway K, Wilks R. Motivational interviewing and cognitive behaviour therapy in the treatment of adolescent overweight and obesity: study design and methodology. *Contemp Clin Trials*. 2008;29(3):359-375. doi:10.1016/j.cct.2007.09.001
2. Brennan L, Walkley J, Wilks R, Fraser SF, Greenway K. Physiological and behavioural outcomes of a randomised controlled trial of a cognitive behavioural lifestyle intervention for overweight and obese adolescents. *Obes Res Clin Pract*. 2013;7(1):e23-e41. doi:10.1016/j.orcp.2012.02.010
3. Brennan L, Wilks R, Walkley J, Fraser SF, Greenway K. Treatment acceptability and psychosocial outcomes of a randomised controlled trial of a cognitive behavioural lifestyle intervention for overweight and obese adolescents. *Behav Chang*. 2012;29(1):36-62. doi:10.1017/bec.2012.5
4. DeBar LL, Stevens VJ, Perrin N, et al. A primary care–based, multicomponent lifestyle intervention for overweight adolescent females. *Pediatrics*. 2012;129(3):e611-e620. doi:10.1542/peds.2011-0863
5. Estabrooks PA, Shoup JA, Gattshall M, Dandamudi P, Shetterly S, Xu S. Automated telephone counseling for parents of overweight children: a randomized controlled trial. *Am J Prev Med*. 2009;36(1):35-42. e2. doi:10.1016/j.amepre.2008.09.024
6. Follansbee-Junger K, Janicke DM, Sallinen BJ. The influence of a behavioral weight management program on disordered eating attitudes and behaviors in children with overweight. *J Am Diet Assoc*. 2010;110(11):1653-1659. doi:10.1016/j.jada.2010.08.005
7. Janicke DM, Sallinen BJ, Perri MG, et al. Sensible treatment of obesity in rural youth (STORY): design and methods. *Contemp Clin Trials*. 2008;29(2):270-280. doi:10.1016/j.cct.2007.05.005
8. Jansen E, Mulkens S, Jansen A. Tackling childhood overweight: treating parents exclusively is effective. *Int J Obes*. 2011;35(4):501-509. doi:10.1038/ijo.2011.16
9. Saelens BE, Sallis JF, Wilfley DE, Patrick K, Cella JA, Buchta R. Behavioral weight control for overweight adolescents initiated in primary care. *Obes Rev*. 2002;10(1):22-32. doi:10.1038/oby.2002.4

Supplementary File S5: Intervention Strategy Components by Intervention Arm for Verified Trials.

Cluster	Components	Frequency in intervention arms (%) k=54
**Intervention Intent, Framing and Outcomes**		
Framing of the intervention (communication strategies)	Education provided on obesity as a disease	8 (15)
	Education that weight loss is required to improve health outcomes	18 (33)
	Education that health outcomes are not dependent on weight	15 (28)
	Education that health behaviours are linked to health outcomes	22 (41)
	Feedback on change in metabolic health outcomes (e.g., insulin sensitivity, cholesterol levels)	7 (13)
	**Interventions with any strategy in this cluster**	**25 (46)**
Outcome related strategies	Encourages weight focused goals	33 (61)
	Discourages weight-focused goals (instead focused on health-related goals)	9 (17)
	Feedback on weight change during the intervention	33 (61)
	Feedback on other measures of weight adiposity (e.g. body composition, waist circumference)	9 (17)
	Promotes weight loss rewards or incentives	16 (30)
	Encourages self-monitoring of weight (e.g. self-weighing at home)	19 (35)
	Discourages home weighing or frequent weighing	10 (19)
	**Interventions with any strategy in this cluster**	**41 (76)**
**Dietary strategies**		
Nutrition education	Education on portion size (e.g. portion plate model, serve sizes, etc)	40 (74)
	Education on label reading	33 (61)
	Education on metabolism	13 (24)
	Education on healthy eating guide (e.g. promotes balanced meals and food groups)	48 (89)
	Education on energy/ macronutrient (e.g. fat, sugar) content of foods	36 (67)
	Categorisation of foods as good versus bad (e.g. traffic light system; defines foods as good vs bad (e.g treat/ sometimes foods)	26 (48)

	Provides cultural adaptations relating to diet	23 (43)
	**Interventions with any strategy in this cluster**	**51 (94)**
Dietary self-monitoring	Dietary self-monitoring – food based (e.g. food diary, points system)	32 (59)
	Dietary self-monitoring – energy based (e.g. calorie counting)	24 (44)
	Dietary self-monitoring – weighing food	8 (15)
	Review/feedback on self-monitoring (e.g., feedback on food diary)	30 (56)
	**Interventions with any strategy in this cluster**	**37 (69)**
Dietary prescription	Hypocaloric diet (reduced calorie diet)	26 (48)
	Traffic light diet (categorising foods as red, yellow, green)	16 (30)
	Intermittent energy restriction/ intermittent fasting (chrononutrition)	3 (6)
	Macronutrient prescription (e.g. low-carbohydrate, high-protein)	8 (15)
	Ketogenic diet	0 (0)
	Very low energy diet (VLED/ VLCD - restrictive calorie restriction e.g. 800-1000 kcal/ day)	2 (4)
	**Interventions with any strategy in this cluster**	**35 (65)**
Delivery of dietary intervention	Prescriptive/ specific meal plan (external control)	10 (19)
	Flexible meal plan (provides choice, ownership over dietary intake)	30 (56)
	Use of meal replacement products – partial or full	2 (4)
	Promotes ‘free’ foods, ad-lib intake of certain foods	3 (6)
	**Interventions with any strategy in this cluster**	**32 (59)**
Dietary behaviour change strategies	Problem solving barriers to dietary change	43 (80)
	Feedback on dietary behaviours (e.g. diet history at visits)	30 (56)
	Encourages dietary focused goals with/ without review	41 (76)
	Shopping support (planning, product choice, family/ partner involvement in food purchases)	25 (46)
	Addresses home/food environment (e.g. identifying triggers, permissive vs restrictive environment; stimulus control)	36 (67)
	Addresses food/meal preparation skills (e.g. cooking demonstrations, recipes)	18 (33)
	**Interventions with any strategy in this cluster**	**50 (93)**
**Eating behaviours/disordered eating**		
Addresses disordered eating	Identifies disordered eating behaviours (e.g. binge eating, emotional eating, secret eating, guilt related to eating, loss of control over eating)	29 (54)

	Explores individual underlying causes/ drivers of disordered eating (e.g. teasing/ bullying, trauma, body image disturbance/pre-occupation with weight and shape, emotional regulation)	12 (22)
	Addresses disordered eating behaviours and cognitions (e.g. identifying triggers, strategies to prevent emotional eating, over-focus on energy expenditure)	23 (43)
	Education on risk of eating disorders	10 (19)
	**Interventions with any strategy in this cluster**	**33 (61)**
Promotes healthful/helpful eating behaviours	Promotes mealtime routines (e.g. regular meals, avoid meal skipping)	30 (56)
	Promotes meal time support (e.g. support while eating, family meals, social eating)	28 (52)
	Addresses meal time environment	22 (41)
	Increasing awareness of hunger/ fullness/ satiety	26 (48)
	Encourages mindful eating principles or practice (e.g. avoiding distractions while eating)	19 (35)
	Encourages intuitive eating principles or practice (e.g. Promotes anti-diet, hunger and fullness, food enjoyment, body respect)	2 (4)
	**Interventions with any strategy in this cluster**	**42 (78)**
**Movement and sleep related strategies**		
Physical activity education	Education to increase physical activity (e.g. staged introduction of activity, suggested activities)	48 (89)
	Promotes joyful movement and activity	24 (44)
	Encourages strict/ formal activity plan (e.g. gym program)	17 (31)
	Education on Non Exercise Activity Thermogenesis (NEAT) (energy expended during tasks of daily living)	9 (17)
	Provides cultural adaptations relating to physical activity	2 (4)
	**Interventions with any strategy in this cluster**	**48 (89)**
Physical activity prescription	Provides a prescriptive exercise plan	16 (30)
	Provides flexible exercise plan (e.g. suggested activities, encouraging choice)	37 (69)
	Provides supervised group exercise classes/ program	18 (33)
	Provides individual personal training	6 (11)
	**Interventions with any strategy in this cluster**	**42 (78)**
Physical activity monitoring	Self-monitoring of activity (e.g. diary, pedometer)	26 (48)
	External feedback on self-monitoring (e.g., feedback on exercise diary, step count)	26 (48)

	**Interventions with any strategy in this cluster**	**26 (48)**
Behaviour change strategies related to physical activity	Encourage activity focused goals (including time, duration, mode) with/ without review	38 (70)
	Increasing skills to undertake physical activity (e.g. demonstration of activity such as pictures/ videos/ live demos, help with scheduling/ planning for activity)	23 (43)
	Feedback on physical activity behaviours and/ or change in fitness	25 (46)
	Problem solving barriers to physical activity	33 (61)
	**Interventions with any strategy in this cluster**	**41 (76)**
Addressing sedentary time	Education to reduce/ limit sedentary time (e.g. screen time)	29 (54)
	Sedentary time focused goals (time, duration, mode) with/ without review	21 (39)
	Encourages self-monitoring of sedentary time (e.g. screen time monitoring, setting app limits, reminders to stand)	19 (35)
	Problem solving barriers to reducing sedentary time	20 (37)
	**Interventions with any strategy in this cluster**	**29 (54)**
Addressing sleep health	Education on sleep health (e.g. duration, quality, routine)	13 (24)
	Sleep health focused goals with/without review	3 (6)
	Encourages self-monitoring of sleep (e.g. sleep diary)	4 (7)
	Problem solving barriers to improving sleep health	10 (19)
	**Interventions with any strategy in this cluster**	**13 (24)**
**Psychosocial health related strategies**		
Addresses mental health conditions	Identifies mental health condition	7 (13)
	Provides referral for psychological support	7 (13)
	Addresses mental health condition within the intervention	3 (6)
	Addresses self-esteem	7 (13)
	**Interventions with any strategy in this cluster**	**9 (17)**
Addresses body image	Addresses body image concerns	17 (31)
	Education on the role of social media (e.g. media literacy training)	7 (13)
	Promotes body compassion/ acceptance/ positivity	12 (22)
	**Interventions with any strategy in this cluster**	**20 (37)**
Addresses weight stigma	Education and/ or strategies to increase resilience to weight stigma, bullying, teasing	13 (24)
	Addresses weight-focused communication skills (e.g. how to communicate with peers/ family about weight, how to address weight-related comments from peers/ family)	8 (15)

	Education to support network persons on weight stigma/teasing	10 (19)
	**Interventions with any strategy in this cluster**	**16 (30)**
Psychosocial health related monitoring	Self-monitoring of thoughts, feelings, mood (e.g. mood diary)	6 (11)
	Review/feedback on self-monitoring (e.g. mood diary)	3 (6)
	Encourages self-assessment of overall wellbeing (e.g. reflective practice)	1 (2)
	**Interventions with any strategy in this cluster**	**6 (11)**
Behaviour change strategies related to psychosocial issues	Encourages psychosocial health related goals with/ without review	7 (13)
	Increases skills to manage psychosocial health (e.g. stress management)	17 (31)
	Inclusion of peer/ social support strategies	21 (39)
	**Interventions with any strategy in this cluster**	**32 (59)**
